# Hypobaric hypoxia can lead to an increase in lung dendritic cells and promote T-cell immunosuppression, thereby preventing the excessive progression of high-altitude pulmonary edema

**DOI:** 10.3389/fimmu.2026.1752864

**Published:** 2026-03-24

**Authors:** Xuemei Wei, Wenhui Shi, Xiang Dong, Weina Kong, Lingling Dong, Ling Zhang, Aziguli Maimaitituerxun, Congying Hou, Dewei Li, Jiangwei Liu, Xiumin Ma

**Affiliations:** 1 Clinical Laboratory Center, Tumor Hospital Affiliated to Xinjiang Medical University, Urumqi, China; 2 Center of Respiratory and Critical Care Medicine, People’s Hospital of Xinjiang Uygur Autonomous Region, Urumqi, China; 3 State Key Laboratory of Pathogenesis, Prevention and Treatment of High Incidence Diseases in Central Asia, Xinjiang Medical University, Urumqi, China; 4 Key Laboratory of Special Environmental Medicine of Xinjiang, General Hospital of Xinjiang Military Command, Urumqi, China

**Keywords:** CD4, dendritic cells, HAPE, hypoxia hypobaric, TNF-α

## Abstract

**Background:**

More and more studies have shown that the immune system regulates the body’s response to hypoxic stress. Immune dysregulation may increase vascular permeability, leading to edema and tissue damage, thereby causing high-altitude pulmonary edema (HAPE). Here, we found an increase in lung dendritic cells (DCs) in rat and mouse models of HAPE, but its specific role in HAPE remains unclear.

**Methods:**

In this study, the function of rat lung DCs and its impact on T cells were analyzed through single-cell sequencing, flow cytometry and *in vitro* co-culture. Then, we confirmed its effects on DCs and T cells by intraperitoneal injection of TNF-α or by using TNF-α -deficient mice. Finally, we evaluated the role of CD4^+^ T cells in the progression of HAPE by eliminating CD4 in mice with neutralizing antibodies.

**Results:**

We demonstrated that hypobaric hypoxia (HH) induced the recruitment of pulmonary DCs in rats and promoted the immunosuppression of T cells (especially CD4^+^ T cells). By injecting TNF-α into rats, we found that the number of DC and CD4^+^CD25^+^ T cells in the lung of HH rats slightly decreased. Interestingly, *in vivo* injection of TNF-α actually led to the production of less TNF-α by CD4^+^ T cells in the lung of HH rats. In the HAPE mouse model, the proportion and the number of DC in the lung of TNF-α -deficient mice were significantly increased, and the level of IL-6 production was significantly decreased. Furthermore, the proportion of CD4^+^CD25^+^ T cells in the lung of TNF-α -deficient mice increased significantly. By eliminating CD4 in mice with neutralizing antibodies, we found that CD4^+^ T cells play a protective role in HAPE.

**Conclusions:**

Our results indicate that hypoxia induces the recruitment of DCs in the lung and mediates the differentiation of T cells into immunosuppressive phenotypes in HAPE rat and mouse models, thereby delaying the progression of HAPE. These findings highlight the potential approach of using immune regulation as a treatment for HAPE.

## Introduction

High altitude pulmonary edema (HAPE) is a serious altitude disease that can occur when an individual rapidly climbs from a plain area to an altitude exceeding 2,500 m (1). It is acute mountain sickness and is non-cardiogenic in nature (2). The clinical symptoms of HAPE are varied, including shortness of breath, pink foamy sputum, and cyanosis (3). At present, the main treatments for HAPE are physical therapy (e.g., descent to low altitude and supplemental oxygen) and medication (e.g., nifedipine and furosemide). Although these measures have greatly reduced HAPE, physical means are not suitable for all situations. In addition, the medicine treatment effect is very limited, some patients will have more serious side effects (4). Therefore, there is an urgent need to find new therapies that have the potential to alleviate HAPE more effectively.

Existing studies have reported that changes in alveolar-capillary barrier permeability caused by pulmonary vasoconstriction and high capillary pressure are considered to be important factors in the occurrence of HAPE (5). Invasive and non-invasive echocardiography also showed that pulmonary hypertension and hypoxia were strongly associated with the development of HAPE (6, 7). However, pulmonary hypertension alone does not cause the occurrence of HAPE, suggesting that the disease is synergistic with multiple factors (8).

Earlier studies have shown that HAPE is not associated with inflammation (7). However, as research continues, a large body of evidence suggests that inflammation may induce or aggravate HAPE (2, 9–12). IL-1β, IL-6 and TNF-α are elevated in HAPE, which is considered to be an important indicator of HAPE occurrence (13). Meanwhile, signaling pathways related to inflammation and hypoxia are activated in HAPE (9). Hypoxia has also been shown to be an integral component in the tumor microenvironment, which drives the inflammatory response by activating hypoxia-inducible factor 1-alpha (HIF-1) and nuclear factor kappa B (NF-κB) signaling pathways (14, 15). These results suggest that elevated inflammatory factors in HAPE are associated with hypoxia, but this mechanism is unclear.

Dendritic cells (DCs) are the most powerful professional antigen presenting cells in the body, which play a key role in the immune response (16). According to different functions, DC can be divided into plasma cell-like dendritic cells (pDCs) and classical dendritic cells (cDCs) (17). cDC can be further divided into cDC1 and cDC2, which are responsible for activating CD8 and CD4^+^ T cells, respectively (18). Studies have shown that continuous hypoxia induces the robust accumulation of leukocytes in the lungs (19). Among them, DCs are the main components of the increase in leukocytes (19). In a mouse model of hypoxic-induced pulmonary hypertension (PH), cDC2 was increased and activated in the lungs (20). PH can be significantly relieved after depletion of cDCs (20). In addition, feeding the mice a high-soluble fiber diet inhibited the development of PH, which is associated with a reduction in DCs (21). Furthermore, another study found that hypoxia can restrict T-cell autoimmunity by activating the HIF-1α pathway in DCs (22). These studies suggest that hypoxic DCs may play a double-edged sword role in pulmonary inflammatory diseases.

To clarify the role of DCs in HAPE, through rat models and *in vitro* experiments, we confirmed that DCs is elevated in the lung of HH rats and exerts immunosuppressive effects. Moreover, we also found that DCs in the lung tissue of HH rats induced CD4^+^ T cells to produce more IL-10 and less TNF-α. As the overall level of TNF-α increases, the TNF-α produced by CD4^+^ T cells decreases accordingly. By introducing TNF-α -deficient mice, we found that the proportion and the absolute number of DCs in the lung tissue of TNF-α -deficient mice significantly increased, and the proportion of CD4^+^CD25^+^ T cells also increased accordingly. Finally, we also confirmed that CD4^+^ T cells have a protective effect in HAPE. In conclusion, our research indicates that DCs is recruited in the lung of HH rats and mice, and induces CD4^+^ T cell immunosuppression, thereby preventing the aggravation of HAPE disease.

## Methods

### Animals

200-250g male sprague dawley (SD) rats and 18-22g male C57BL/6 wild-type (WT) mice were purchased from Experimental Animal Center of Xinjiang Medical University [SYXK (New) 2016-0003]. C57BL/6 TNF-α-knockout (KO) mice (005540) were purchased from the Jackson Laboratory in the United States. The rats and mice were raised in the pathogen-free environment with a 12-hour light/dark cycle in the animal experimental center of Xinjiang Medical University. All the procedures and experiments were approved by the Experimental Animal Ethics Committee of Xinjiang Medical University (No: 20240514-185).

### High altitude pulmonary edema

The HAPE rats was established by using the Simulated Climate Cabin for the Special Environment of Northwest China, with the experimental conditions as described before (23). For rats, they were placed in a hypobaric hypoxic (HH) environment for 3 days to induce HAPE. For mice, they were placed in a hypobaric hypoxic environment for 3 days or 2 weeks to induce HAPE. The modeling effect of HAPE was evaluated by observing the changes in lung section pathological staining, lung weight, lung coefficient and lung dry/wet weight ratio. The calculation method of lung coefficient: whole lung weight/rat or mouse body weight × 100%.

### Single-cell sequencing of rat lung tissue

#### Lung tissue collection and processing

In order to obtain better single-cell sequencing data, we selected samples by calculating rat lung coefficients and lung weights. First, the rats were euthanized, the lung tissue was separated aseptically, and the total lung weight was recorded after removing the connective tissue on the lung tissue surface. Then, the left lung was separated and cut into small pieces, washed with PBS and placed in the sCelLive^®^ Tissue Preservation Solution (Singleron).

For the control group, 3 samples with similar lung weights and lung coefficients were selected for further sequencing. For the hypobaric anoxic group, samples with significantly higher total lung weight and lung coefficient than the control group were labeled. Then, 3 samples were screened according to the previous method for sequencing.

#### Tissue dissociation and preparation

The specimens were washed with Hanks Balanced Salt Solution (HBSS) for three times, and then digested with 3 mL sCelLive^®^ Tissue Dissociation Solution (Singleron) by Singleron PythoN^®^ Tissue Dissociation System at 37°C for 15 min. The cell suspension was collected and filtered through a 40-micron sterile strainer. Afterwards, the GEXSCOPE^®^ red blood cell lysis buffer (RCLB, Singleron) was added, and the mixture [Cell: RCLB = 1:2 (volume ratio)] was incubated at room temperature for 5–8 min to remove red blood cells. The mixture was then centrifuged at 300 × g 4°C for 5 mins to remove supernatant and suspended softly with PBS.

Finally, the samples were stained with Trypan Blue and the cell viability was evaluated microscopically.

#### Single cell RNA sequencing

Single-cell suspensions were converted to barcoded scRNA-seq libraries by using the Chromium Single Cell Library, Gel Bead & Multiplex Kit (10x Genomics), and following the manufacturer’s instructions. Briefly, cells were partitioned into Gel Beads in Emulsion in the ChromiumTM Controller instrument where cell lysis and barcoded reverse transcription of RNA occurred. Libraries were prepared using 10x Genomics Library Kits and sequenced on Illumina NovaSeq X Plu with 150 bp paired end reads. The above steps are completed at Singleron Biotechnology Co., LTD. These data have been uploaded to the NCBI database (http://www.ncbi.nlm.nih.gov/bioproject/1422438).

#### Analyse

Raw data were analyzed using CellRanger software (v5.0; 10X Genomics) to generate quality indicators, as well as per gene count data for each cell. To achieve normalization and batch correction, R statistical environment was used to analyze and visualize the scRNA-seq data. Subsequently, the data is clustered and annotated.

### Isolation of mononuclear cells from rat lung

The lungs were perfused with HBSS (Thermo Fisher) through the right ventricle, cut into small pieces with scissors, and digested for 1 h at 37°C in RPMI 1640 containing 10% v/v of FBS (Gibco), 1 mg·ml^-1^ collagenase IV (Thermo Fisher), 0.1 mg·ml^-1^ DNase I (Solarbio). Then, the suspension of lung mononuclear cells was obtained by resuspend with PBS for subsequent experiments.

### Flow cytometry

Single-cell suspension was incubated in PBS buffer (PBS containing 0.2% BSA and 0.1% sodium azide) in the presence of mouse serum (for rat) or anti-mouse CD16/32 (for mouse) for 30 min at 4°C prior to staining. For cell surface staining: the prepared antibody was added and incubated at 4°C in the dark for 30 minutes. For intracellular cytokine staining: add the prepared cell activation cooktail (with Brefeldin A) to the cell suspension and incubate it in a cell incubator for 4 hours. Then, according to the manufacturer’s instructions, carry out steps such as cell surface staining, cell perforation, and intracellular cytokine staining. Flow cytometer was performed using an LSRFortessa, and data were analyzed with FlowJo™ v10.6.1 program (Treestar, San Carlos, CA). Information about the antibodies used in this study was listed in **Supplementary Table 1**. For the subpopulations of lung DCs in rats, we classified them according to the following markers (1): cDC1: CD45^+^CD3^-^CD45RA^-^CD11b/c^+^MHCII^+^CD103^+^CD4^-^ (2); cDC2 enriched: CD45^+^CD3^-^ CD45RA^-^CD11b/c^+^MHCII^+^CD103^+^CD4^+^ (3); pDC: CD45^+^CD3^-^CD45RA^-^cDC^-^CD4^+^.

### Purification of DC and T cells from rat lungs

For cell population isolation, a rat T cell isolation kit (130-090-320) was purchased from Miltenyi Biotec, and a rat tissue DCs isolation kit (DC2012RATP) was also bought from Tianjin Haoyang Biological Products Technology Co., LTD.

For DCs isolation, mononuclear cells were isolated from rat lung tissue as previously described. Then, separate the liquid containing DCs according to the manufacturer’s instructions. Finally, CD11b/c and MHC II were added to the liquid containing DCs. After incubation at 4°C for 30 min, the DCs (CD11b/c^+^MHC II^+^) were sorted out by flow cytometry.

T cells were isolated and purified by the immunomagnetic depletion. In brief, rat lung mononuclear cells were isolated. Then, 20 μl of MACS Pan T cell microbeads were added per 10^7 total cells, mixed, and incubated for 30 min on ice. Subsequently, the cell suspension was passed over a column and washed with 2ml of buffer. Effluent cells were CD3^-^ cells; bound cells were CD3^+^ cells. The latter were collected by removing the column from the magnet and eluting it with medium.

### Enzyme-linked immunosorbent assay

The isolated rat lung DCs and T cells were mixed (DCs: T = 5×10^5: 1.5×10^6), and then cultured for 6 h in normoxic or hypoxic (1% O_2_) environments. Then, the cell supernatant was collected and the level of TNF-α, IL-10, IL-6, IL-1β, il-18 was detected using the ELISA kit (Shanghai Jianglai Biotechnology Co., LTD.).

### Untargeted metabolomics

After thawing the co-culture supernatant at room temperature, 100 μL of sample was taken and mixed with 400 μL of extraction solution (MeOH: ACN, 1:1 (v/v)), the extraction solution contain deuterated internal standards, the mixed solution were vortexed for 30 s, sonicated for 10 min in 4 °C water bath, and incubated for 1 h at -40°C to precipitate proteins.

Then the samples ware centrifuged at 12000 rpm for 15 min at 4°C. The supernatant was transferred to a fresh glass vial for analysis. The quality control (QC) sample was prepared by mixing an equal aliquot of the supernatant of samples. All the above steps were completed by Shanghai BIOTREE Biomedical Technology Co., LTD.

### Intraperitoneal injection of TNF-α

This experiment was conducted in accordance with our previous description (23). In brief, on the day before hypobaric hypoxia, each rat was intraperitoneally injected with 10 μg of TNF-α once a day for 4 d.

### Pathological staining

The collected lung tissues of rats or mice were fixed in 4% paraformaldehyde for 48 hrs. Dehydrated, paraffin-embedded lung tissues were into 5-μm sections, which were then de-paraffinized in two changes of xylene for 15 minutes each and then hydrated through a graded series of ethanol (100, 95, 70, 50%) and deionized water, each for 5 minutes. Sections were then stained in hematoxylin and eosin. Finally, the pictures were collected using cellSens Dimension software (Olympus).

### Depletions

CD4^+^ T cell subsets were depleted by administering 400 μg of CD4^+^ T-cells depleting antibody (clone GK1.5, BioXCell) i.p. twice weekly beginning one day prior to hypobaric hypoxia.

### Statistical analysis

Statistical analysis was performed using IBM SPSS Statistics 25. The independent sample t test was used to compare the two groups. All scRNA-seq analyses were performed using R. For all results, *P* < 0.05 was considered to be significant. (p-values were expressed as follows: *p values < 0.05; **p values < 0.01; ***p values < 0.001).

## Results

### Hypobaric hypoxia induces an increase in DC in the rat lung

**Figure 1:**
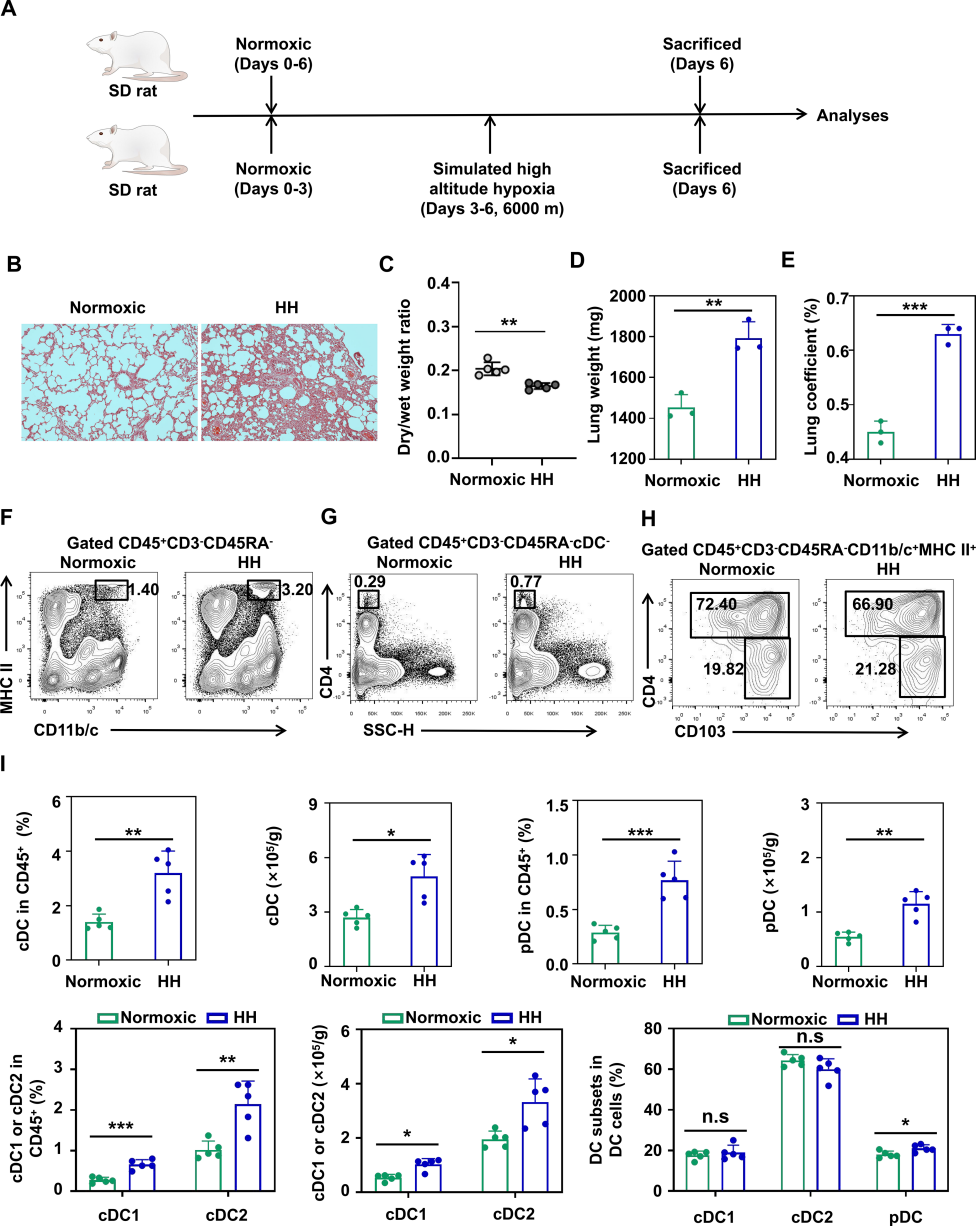
Hypobaric hypoxia induces the accumulation of DCs in the rat lung. **(A)** Establishment of the experimental protocol for the HAPE rat model. **(B)** Representative images of HE staining of lung tissue sections from rats in the normoxic and HH groups. **(C)** Dry/wet weight ratio in the lung of rats in the normoxic and HH groups (5 rats per group). **(D)** Comparison of lung weight between normoxic and hypobaric hypoxic rats (3 rats per group). **(E)** Comparison of lung coefficient between normoxic and hypobaric hypoxic rats (3 rats per group). **(F)** Representative flow cytometry plot of lung cDC in the normoxic and hypobaric hypoxia rats. **(G)** Representative flow cytometry plot of lung pDC in the normoxic and hypobaric hypoxia rats. **(H)** Representative flow cytometry plot of lung cDC1 and cDC2 in the normoxic and hypobaric hypoxia rats. **(I)** Percentage and absolute numbers of lung DC subsets in the normoxic and hypobaric hypoxia rats (5 rats per group). All data are presented as mean ± SD. *P < 0.05, **P < 0.01, ***P < 0.001, n.s., *P* > 0.05.

We established a rat HAPE model through a Simulated Climate Cabin for the Special Environment of Northwest China (**Figure 1A**). Pathological staining and the ratio of dry/wet lung weight confirmed that this method for modeling is reliable and stable (**Figures 1B, C**). Then, we repeated the model and selected three rats from the hypobaric hypoxia group (HH), whose lung weight and lung coefficient were much higher than normoxia group (**Figures 1D, E**). This part of the rat lung tissue was used by us for single-cell sequencing (**Supplementary Figure 1A**). We identified 12 different types of cells (**Supplementary Figures 1B, C**). To clarify the changes in the DC subpopulations in the HH rat lungs, we further divided DCinto cDC1, cDC2 and pDC (**Supplementary Figures 2A–C**). Moreover, we characterized it using flow cytometry (24) (**Supplementary Figure 2D**, **Figures 1F–H**). We found that the proportions and absolute number of lung cDC (including cDC1 and cDC2) and pDC in the immune cells of HH rats increased significantly (**Figure 1I**). However, the proportion of lung DC subsets in DC in the HH group was nearly the same as the normoxic group (**Figure 1I**).

Collectively, these results demonstrate that hypobaric hypoxia induces an increase in DC in the rat lungs. These DC subsets show a uniform upward trend, indicating that these cells may have been recruited from outside the lungs.

### The lung DCs of HH rats has significantly different gene expression and metabolic characteristics

**Figure 2:**
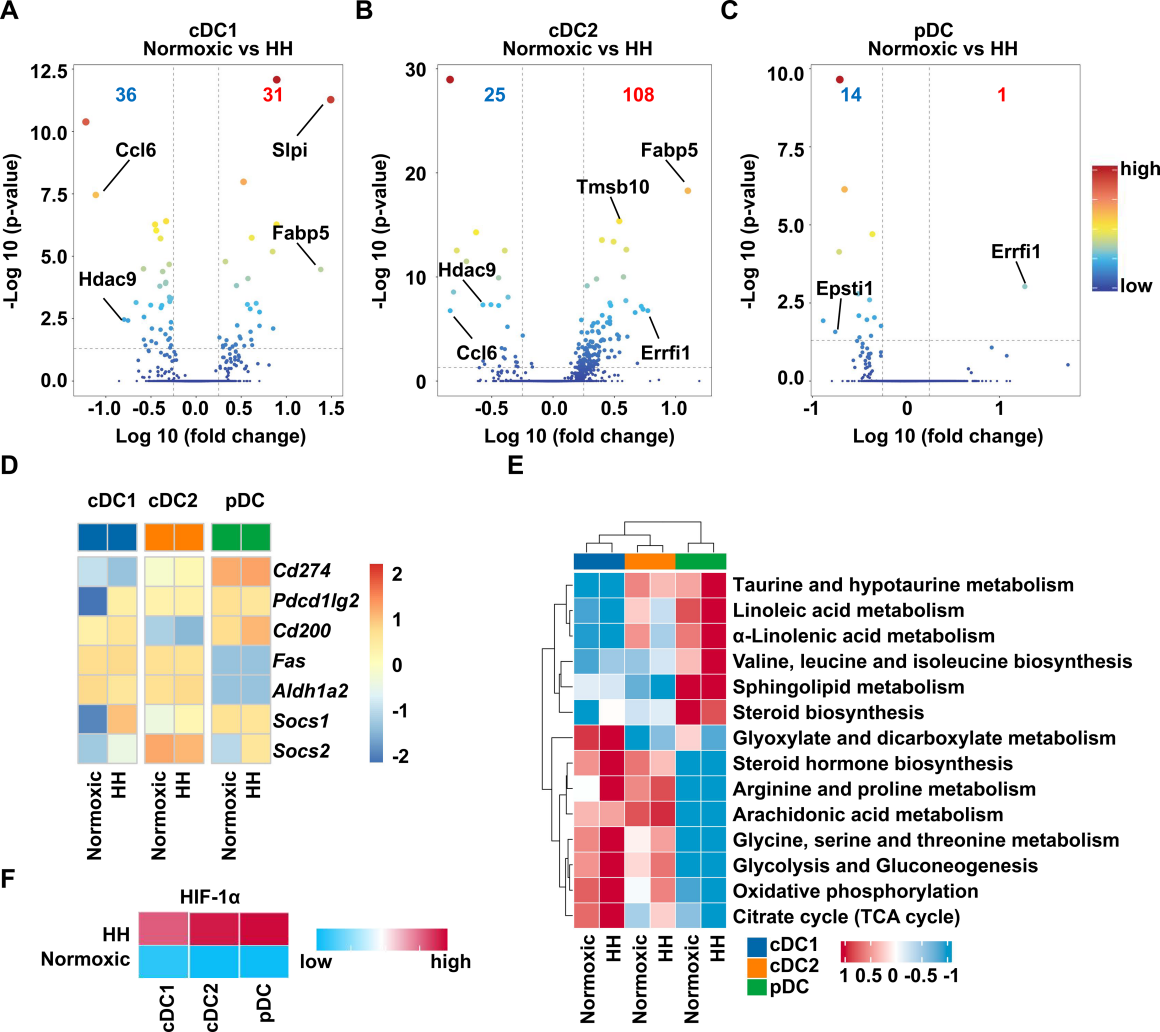
Hypobaric hypoxia induces changes in gene expression and metabolic levels of rat lung DC subsets. **(A)** The volcano plot shows the differential gene expression of cDC1 in the lung from hypobaric hypoxia and normoxic rats. **(B)** The volcano plot shows the differential gene expression of cDC2 in the lung from hypobaric hypoxia and normoxic rats. **(C)** The volcano plot shows the differential gene expression of pDC in the lung from hypobaric hypoxia and normoxic rats. **(D)** Heatmap displaying expression level of genes related to immune regulation in the lung DC subsets of hypobaric hypoxia and normoxic rats. **(E)** Heatmap indicating the metabolic levels in the lung DC subsets from hypobaric hypoxia and normoxic rats. **(F)** Heatmap reflects the expression levels of HIF-1α in the lung DC subsets of hypobaric hypoxia and normoxic rats.

To further investigate the impact of hypobaric hypoxia on DC, we analyzed the differences in gene expression within the DC subsets. The results showed that hypobaric hypoxia induced down-regulation of gene expression related to promoting inflammation (*Ccl6, Hdac9, Epsti1*) in the DC subsets (25–27) (**Figures 2A–C**, **Supplementary Tables 2–4**). Moreover, the genes related to inhibiting inflammation (*Slpi, Fabp5, Tmsb10, Errfi1*) were upregulated in the HH group (28–31) (**Figures 2A–C**, **Supplementary Tables 2–4**). Subsequently, we also analyzed the expression levels of immunosuppressive molecules in the DC subsets. We found that the expressions of molecules (e.g., Cd274, Pdcd1lg2, Cd200, Socs1, Socs2) in the DC subsets of the HH group were increased to varying degrees (**Figure 2D**). In addition, the expression levels of genes related to DCs maturation, migration and Th2response have also changed (**Supplementary Figure 3**).

Considering the significance of metabolism in hypoxia, we analyzed the metabolic pathway levels related to amino acids, lipids and carbohydrates in the DC subgroup. Interestingly, cDC1, cDC2 and pDC have completely different metabolic characteristics (**Figure 2E**). After hypobaric hypoxia, the levels of most amino acid, lipid and carbohydrate metabolic pathways were increased in DCs (**Figure 2E**). Since the HIF-1α pathway plays an important role in hypoxia, we also analyzed its expression in DCs. The results indicated that the expression of HIF-1α was increased in the DCs of the HH group (**Figure 2F**).

Collectively, these results indicate that hypobaric hypoxia induces the activation of amino acid, lipid and carbohydrate metabolic pathways in rat lung DCs, as well as an increase in HIF-1α expression, thereby enabling DCS to exert immunosuppressive effects.

### HH induces immunosuppression of lung T cells of rats

T cells and DCs engage in a tightly coordinated interaction, thereby regulating immune responses(32). To further understand whether DCs can regulate theimmune response of T cells in a hypobaric hypoxic environment, we examined the phenotype and function of T cells (**Supplementary Figure 4**). We found that there was no significant difference in the percentage of lungCD3^+^ T cells in the HH rats, while the absolute number showed an increasing trend (**Supplementary Figure 5A**). Further analysis revealed that the increased cells were CD4^+^ T cells (**Supplementary Figures 5B, C**). Then, we found that the percentage and absolute number of CD4^+^CD25^+^T cells in the two groups were not statistically significant (**Supplementary Figures 5D, E**). Moreover, the proliferation ability of CD4^+^CD25^-^ T cells in the HHgroup decreased (**Supplementary Figures 5F, G**). Furthermore, the percentage and absolute number of CD8^+^CD25^+^ T cellsin the two groups were not statistically significant, but the proliferation ability ofCD8^+^CD25^-^ T cells in the HH group decreased (**Supplementary Figures 5H–K**).

**Figure 3:**
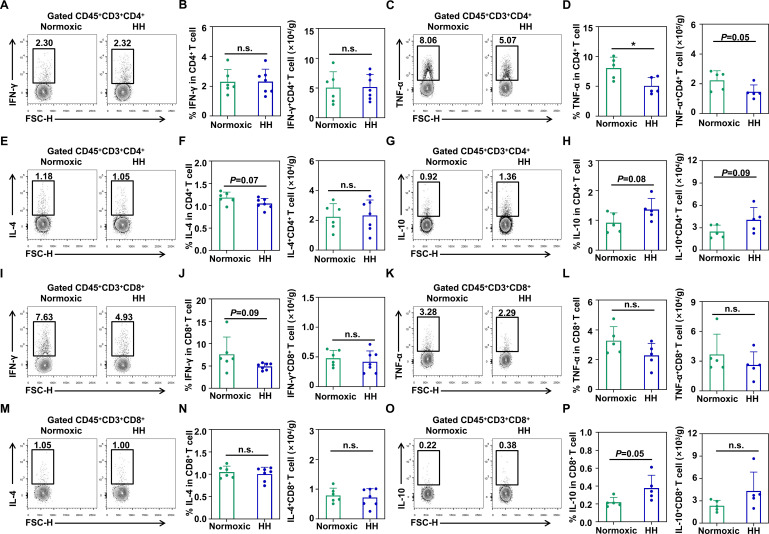
Hypobaric hypoxia induces immunosuppression of CD4^+^ T cells in the rat lung. **(A)** Representative flow cytometry plot of IFN-γ production by CD4^+^ T cells in the lung from normoxic and hypobaric hypoxia rats. **(B)** Percentage and absolute numbers of IFN-γ production by CD4^+^ T cells in the lung from normoxic and hypobaric hypoxia rats (6–7 rats per group). **(C)** Representative flow cytometry plot of TNF-α production by CD4^+^ T cells in the lung from normoxic and hypobaric hypoxia rats. **(D)** Percentage and absolute numbers of TNF-α production by CD4^+^ T cells in the lung from normoxic and hypobaric hypoxia rats (5 rats per group). **(E)** Representative flow cytometry plot of IL-4 production by CD4^+^ T cells in the lung from normoxic and hypobaric hypoxia rats. **(F)** Percentage and absolute numbers of IL-4 production by CD4^+^ T cells in the lung from normoxic and hypobaric hypoxia rats (6–7 rats per group). **(G)** Representative flow cytometry plot of IL-10 production by CD4^+^ T cells in the lung from normoxic and hypobaric hypoxia rats. **(H)** Percentage and absolute numbers of IL-10 production by CD4^+^ T cells in the lung from normoxic and hypobaric hypoxia rats (5 rats per group). **(I)** Representative flow cytometry plot of IFN-γ production by CD8^+^ T cells in the lung from normoxic and hypobaric hypoxia rats. **(J)** Percentage and absolute numbers of IFN-γ production by CD8^+^ T cells in the lung from normoxic and hypobaric hypoxia rats (6–7 rats per group). **(K)** Representative flow cytometry plot of TNF-α production by CD8^+^ T cells in the lung from normoxic and hypobaric hypoxia rats. **(L)** Percentage and absolute numbers of TNF-α production by CD8^+^ T cells in the lung from normoxic and hypobaric hypoxia rats (5 rats per group). **(M)** Representative flow cytometry plot of IL-4 production by CD8^+^ T cells in the lung from normoxic and hypobaric hypoxia rats. **(N)** Percentage and absolute numbers of IL-4 production by CD8^+^ T cells in the lung from normoxic and hypobaric hypoxia rats (6–7 rats per group). **(O)** Representative flow cytometry plot of IL-10 production by CD8^+^ T cells in the lung from normoxic and hypobaric hypoxia rats. **(P)** Percentage and absolute numbers of IL-10 production by CD8^+^ T cells in the lung from normoxic and hypobaric hypoxia rats (5 rats per group). All data are presented as mean ± SD. *P < 0.05, n.s., *P* > 0.05.

Subsequently, we also investigated the ability of T cells to secrete cytokines. We observed that compared with the lung CD4^+^ T cells in the normoxia rats, there was no difference in the level of IFN-γ produced by CD4^+^ T cells in the HH rats (**Figures 3A, B**). Moreover, the lung CD4^+^ T cells from HH rats produced less TNF-α and IL-4, while more IL-10 (**Figures 3C–H**). In addition, in the HH rat lungs, the level of IFN-γ produced by CD8^+^ T cells decreased, while there was no statistically significant in the levels of TNF-α and IL-4 (**Figures 3I–N**). Similar to CD4^+^ T cells, hypobaric hypoxia induces more IL-10 production in rat lung CD8^+^ T cells (**Figures 3O, P**). However, there was no statistically significant in the absolute number of IFN-γ^+^ and IL-10^+^ CD8^+^ T cells in the lungs of HH rats (**Figures 3J, P**).

In conclusion, hypobaric hypoxia reduces the production of pro-inflammatory factors, while increasing the production of anti-inflammatory factors in lung T cells of rats.

### Hypoxia DCs induce T-cell immunosuppression

**Figure 4:**
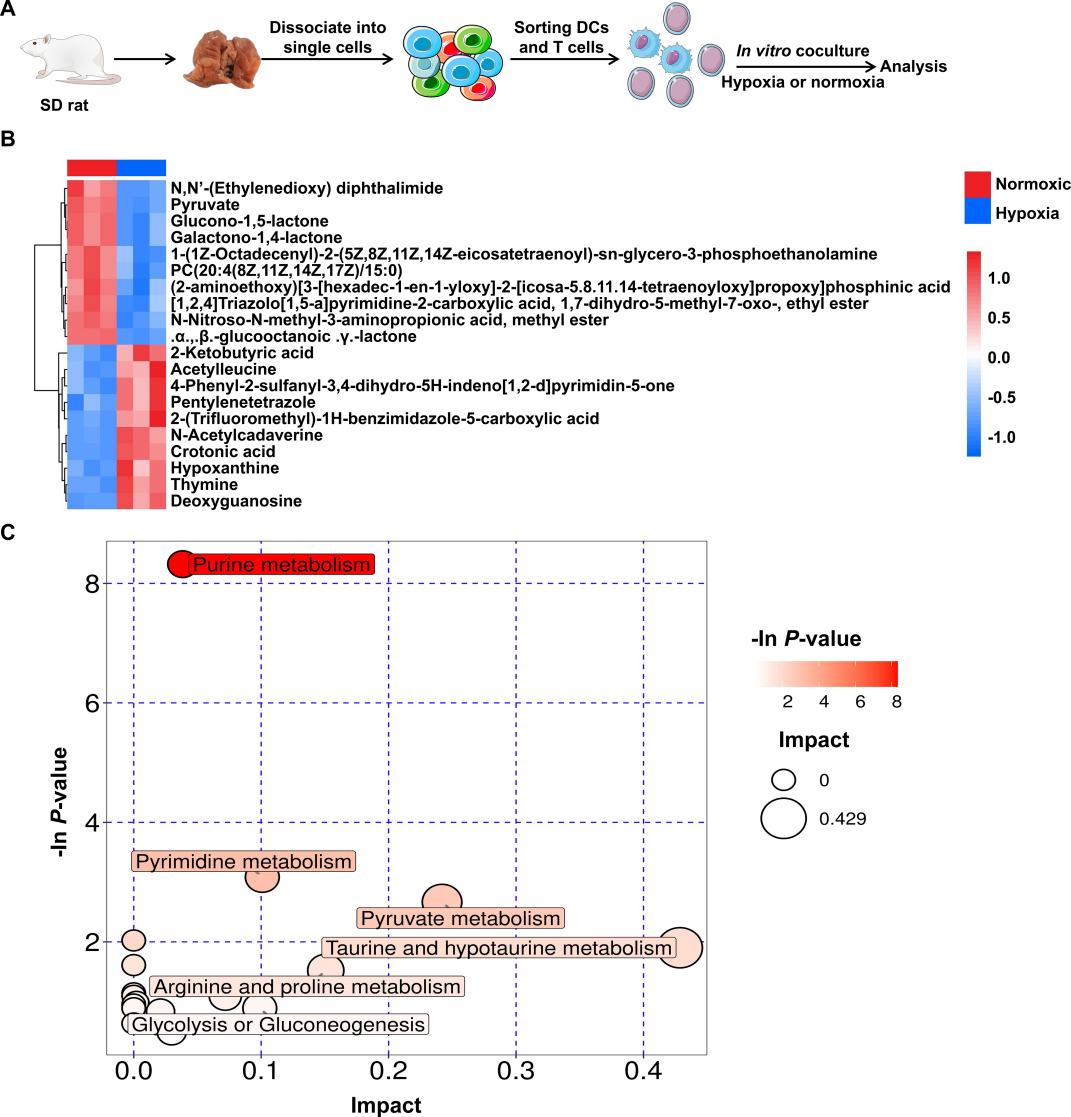
Hypoxia induces metabolic changes in DCs. **(A)** Establishment of an *in vitro* co-culture system of DC and T cells. **(B)** Untargeted metabolomics analysis showed the changes in metabolites in the supernatant of DC and T cells after co-culture for 6 h. **(C)** The bubble chart showed the metabolic pathways involved in the differential metabolites.

Although the function of lung T cells in HH rats is biased towards immunosuppression, it remains unclear whether their differentiation is mediated by DCs. To confirm the effect of DCs on T cell differentiation, we sorted rat lung DCs and T cells and co-cultured them under normoxic or hypoxic (1% O_2_) conditions (**Figure 4A**). After hypoxia, the levels of TNF-α, IL-1β and IL-18 in the supernatant of theco-culture significantly decreased, while the level of IL-10 significantly increased, confirmingthat hypoxia induced immunosuppression of DCs and T cells (**Supplementary Figure 6**). Furthermore, untargeted metabolomics analysis indicated that the metabolic levels in the culture supernatant changed significantly after hypoxia (**Figure 4B**, **Supplementary Table 5**). These differential metabolites are mainly involved in purine metabolism, pyrimidine metabolism and pyruvate metabolism (**Figure 4C**).

### TNF-α reduces lung DCs and inhibits the production of TNF-α^+^ CD4^+^ T cells in HH rats

**Figure 5:**
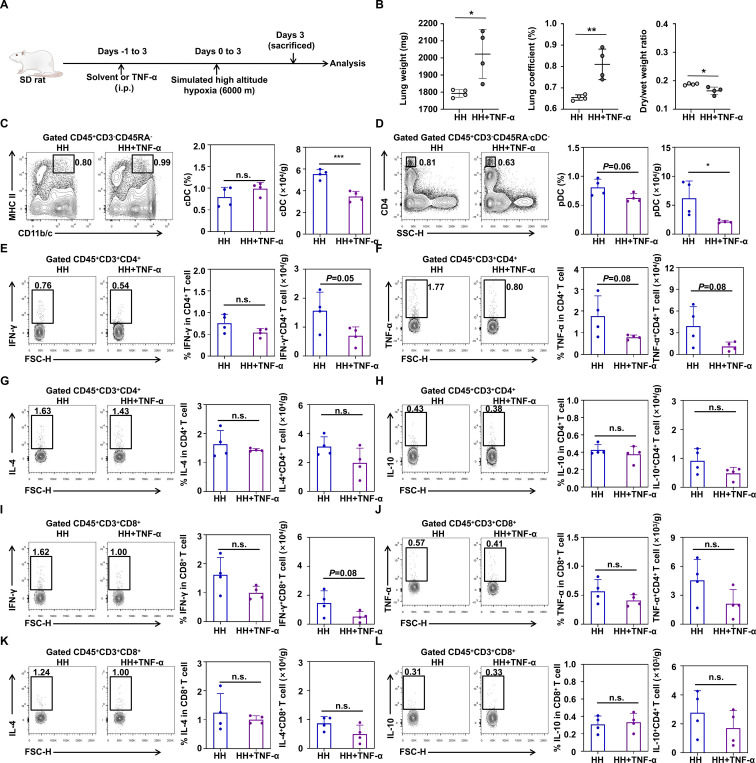
The increase in the level of TNF-α *in vivo* leads to a decrease in the level of TNF-α produced by CD4^+^ T cells. **(A)** Establishment of the protocol for injecting TNF-α into HH rats. **(B)** Comparison of lung weight, lung coefficient and dry/wet weight ratio between HH and HH+TNF-α rats (4 rats per group). **(C)** Representative flow cytometry plot, percentage and absolute numbers of lung cDC from the hypobaric hypoxia and hypobaric hypoxia+TNF-α rats (4 rats per group). **(D)** Representative flow cytometry plot, percentage and absolute numbers of lung pDC from the hypobaric hypoxia and hypobaric hypoxia+TNF-α rats (4 rats per group). **(E)** Representative flow cytometry plot, percentage and absolute numbers of IFN-γ production by CD4^+^ T cells in the lung from hypobaric hypoxia and hypobaric hypoxia+TNF-α rats (4 rats per group). **(F)** Representative flow cytometry plot, percentage and absolute numbers of TNF-α production by CD4^+^ T cells in the lung from hypobaric hypoxia and hypobaric hypoxia+TNF-α rats (4 rats per group). **(G)** Representative flow cytometry plot, percentage and absolute numbers of IL-4 production by CD4^+^ T cells in the lung from hypobaric hypoxia and hypobaric hypoxia+TNF-α rats (4 rats per group). **(H)** Representative flow cytometry plot, percentage and absolute numbers of IL-10 production by CD4^+^ T cells in the lung from hypobaric hypoxia and hypobaric hypoxia+TNF-α rats (4 rats per group). **(I)** Representative flow cytometry plot, percentage and absolute numbers of IFN-γ production by CD8^+^ T cells in the lung from hypobaric hypoxia and hypobaric hypoxia+TNF-α rats (4 rats per group). **(J)** Representative flow cytometry plot, percentage and absolute numbers of TNF-α production by CD8^+^ T cells in the lung from hypobaric hypoxia and hypobaric hypoxia+TNF-α rats (4 rats per group). **(K)** Representative flow cytometry plot, percentage and absolute numbers of IL-4 production by CD8^+^ T cells in the lung from hypobaric hypoxia and hypobaric hypoxia+TNF-α rats (4 rats per group). **(L)** Representative flow cytometry plot, percentage and absolute numbers of IL-10 production by CD8^+^ T cells in the lung from hypobaric hypoxia and hypobaric hypoxia+TNF-α rats (4 rats per group). All data are presented as mean ± SD. *P < 0.05, **P < 0.01, ***P < 0.001, n.s., *P* > 0.05.

The level of TNF-α may mediate the progression of HAPE (33). However, it remains unclear how TNF-α mediates the development of lung DCs and T cells in HAPE. Here, on the basis of hypobaric hypoxia, we injected TNF-α into the rats through intraperitoneal injection (**Figure 5A**). We found that TNF-α significantly increased the lung weight and lung coefficient of HH rats, and significantly decreased the lung dry/wet weight ratio (**Figure 5B**). We found that TNF-α reduced lung DCs in HH rats (**Figures 5C, D**). In addition, the absolute number of T cells and their subsets decreased, but thedifference was not statistically significant (**Supplementary Figures 7A–C**). Further analysis revealed that TNF-α reduced the percentage ofCD4^+^CD25^+^ T cells in the HH rat lungs (**Supplementary Figures 7D, E**). Moreover, the absolute number of CD4^+^CD25^+^ T cells decreased, but the difference was not statistically significant (**Supplementary Figures 7D, E**). Consistent with the percentage and absolute number results, the proliferation ability of CD4^+^CD25^+^ T cells was weakened (**Supplementary Figures 7F, G**). The absolute number of CD8^+^CD25^+^ T cells decreased, but the difference was not statistically significant (**Supplementary Figures 7H, I**). Furthermore, TNF-α did not affect the proliferation ability of CD8^+^CD25^+^ and CD8^+^CD25^-^ T cells in HH rat lungs (**Supplementary Figures 7J, K**).

To further clarify the impact of TNF-α on T cell function, we examined the levels of IFN-γ, TNF-α, IL-4 and IL-10 produced by T cell subsets. Interestingly, the increase in total level of TNF-α slightly inhibited the differentiation of IFN-γ^+^ and TNF-α^+^ CD4^+^ T cells, and their absolute numbers also tended to decrease (**Figures 5E, F**). Exogenous TNF-α does not affect the level of IL-4 and IL-10 produced by CD4^+^ T cells (**Figure 5G, H**). Similarly, TNF-α slightly inhibited the production of IFN-γ and TNF-α by CD8^+^ T cells, reduced the absolute number of IFN-γ^+^ and TNF-α^+^ CD8^+^ T cells, but did not affect the production of IL-4^+^ and IL-10^+^ CD8^+^ T cells (**Figures 5I–L**).

Overall, TNF-α can reduce the number of DCs and inhibit the production of CD4^+^CD25^+^ T cells in the HH rat lungs. In terms of function, exogenous TNF-α inhibited the levels of TNF-α produced by CD4^+^ T cell subsets.

### TNF-α deficiency increases lung DCs and promotes the differentiation of CD4^+^CD25^+^ T cells in HH mice

**Figure 6:**
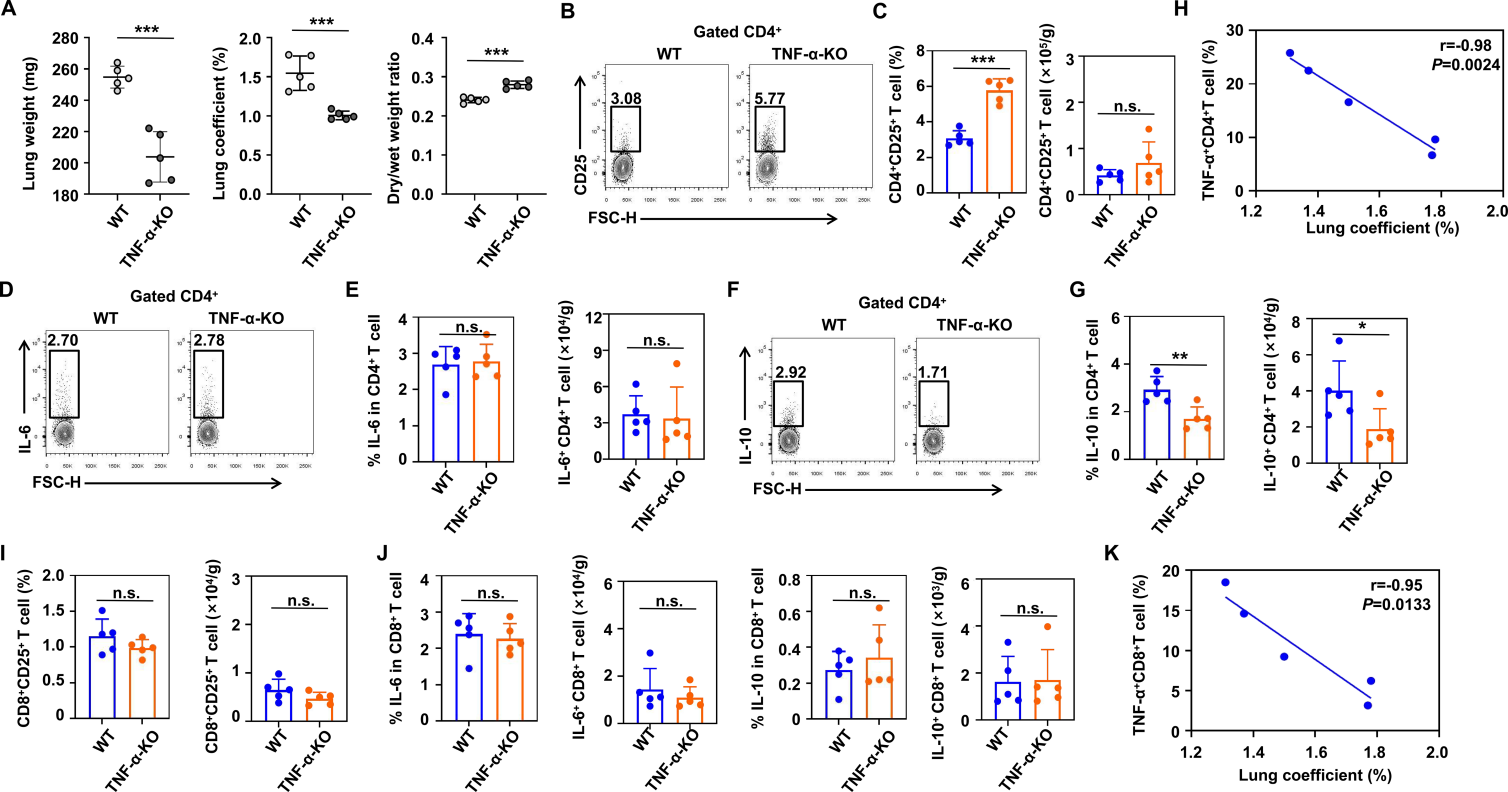
TNF-α deficiency can promote the differentiation of CD4^+^CD25^+^ T cells in the lung of hypobaric hypoxic mice. **(A)** Comparison of lung weight, lung coefficient and dry/wet weight ratio between WT and TNF-α-KO mice after 2 weeks of hypobaric hypoxia (5 mice per group). **(B)** Representative flow cytometry plot of CD4^+^CD25^+^ T cells in the lung from WT and TNF-α-KO mice after 2 weeks of hypobaric hypoxia. **(C)** Percentage and absolute numbers of CD4^+^CD25^+^ T cells in the lung from WT and TNF-α-KO mice after 2 weeks of hypobaric hypoxia (5 mice per group). **(D)** Representative flow cytometry plot of IL-6 production by CD4^+^ T cells in the lung from WT and TNF-α-KO mice after 2 weeks of hypobaric hypoxia. **(E)** Percentage and absolute numbers of IL-6 production by CD4^+^ T cells in the lung from WT and TNF-α-KO mice after 2 weeks of hypobaric hypoxia (5 mice per group). **(F)** Representative flow cytometry plot of IL-10 production by CD4^+^ T cells in the lung from WT and TNF-α-KO mice after 2 weeks of hypobaric hypoxia. **(G)** Percentage and absolute numbers of IL-10 production by CD4^+^ T cells in the lung from WT and TNF-α-KO mice after 2 weeks of hypobaric hypoxia (5 mice per group). **(H)** Correlation between the lung coefficient and the TNF-α production by CD4^+^ T cells from WT mice after 2 weeks of hypobaric hypoxia. **(I)** Percentage and absolute numbers of CD8^+^CD25^+^ T cells in the lung from WT and TNF-α-KO mice after 2 weeks of hypobaric hypoxia (5 mice per group). **(J)** Percentage and absolute numbers of IL-6 and IL-10 production by CD8^+^ T cells in the lung from WT and TNF-α-KO mice after 2 weeks of hypobaric hypoxia (5 mice per group). **(K)** Correlation between the lung coefficient and the TNF-α production by CD8^+^ T cells from WT mice after 2 weeks of hypobaric hypoxia. All data are presented as mean ± SD. *P < 0.05, **P < 0.01, ***P < 0.001, n.s., *P* > 0.05.

To further verify the effects of TNF-α on DCs and T cells in the HAPE model, we introducedTNF-α-deficient mice. We found that the degree of pulmonary edema in mice was more severeafter two weeks of hypobaric hypoxia (**Supplementary Figure 8A**). Subsequently, we established a HAPE model of TNF-α-KO mice (**Supplementary Figure 8B**). The results showed that the lung weight and lung coefficient of TNF-α-KO mice were significantly reduced, while the lung dry-wet weight ratio was significantly increased (**Figure 6A**). In the wild-type (WT) mice lungs, the absolute number of cells was significantly positively correlated with the lung coefficient (**Supplementary Figure 8C**). The percentage of lung CD3^+^ T cells of TNF-α-deficient mice decreased, but there was no statistically significant in the absolute number of cells (**Supplementary Figure 8D**). In addition, when compared with WT mice, there was no statistically significant in the percentage and absolute number of lung CD4 and CD8^+^ T cells of TNF-α-KO mice (**Supplementary Figures 8E, F**). However, we observed that the percentage of lung CD4^+^CD25^+^ T cells of TNF-α-KO mice significantly increased, and the absolute number of cells also slightly rose (**Figures 6B, C**). Interestingly, there was no significant difference in the level and absolute number of IL-6 production by lung CD4^+^ T cells of TNF-α-deficient mice, while the level and absolute number of IL-10 was significantly reduced (**Figures 6D–G**). Correlation analysis indicated that the level of TNF-α produced by lung CD4^+^ T cells of WT mice was significantly negatively correlated with the lung coefficient (**Figure 6H**). Furthermore, we found that the percentage and absolute number of CD8^+^CD25^+^, IL-6^+^CD8^+^, and IL-10^+^CD8^+^ T cells were not statistically significant (**Figures 6I, J**). Correlation analysis also indicated that the level of TNF-α produced by lung CD8^+^ T cells of WT mice was significantly negatively correlated with the lung coefficient (**Figure 6K**).

Next, we analyzed the changes in lung DCs of HH mice after TNF-α deficiency. The results showed that the percentage and absolute number of lung DCs in TNF-α-KO mice increased significantly, while the percentage of IL-6 production decreased significantly (**Supplementary Figures 8G–J**). Moreover, the absolute number of IL-6^+^ DC did not differ significantly (**Supplementary Figure 8J**). Furthermore, the percentage of DCs and IL-6^+^ DC were negatively correlated and positively correlated with the lung coefficient respectively, while the correlation between TNF-α^+^ DC and the lung coefficient was not statistically significant (**Supplementary Figures 8K–M**).

### The depletion of CD4^+^ T cells exacerbated the progression of HAPE

**Figure 7:**
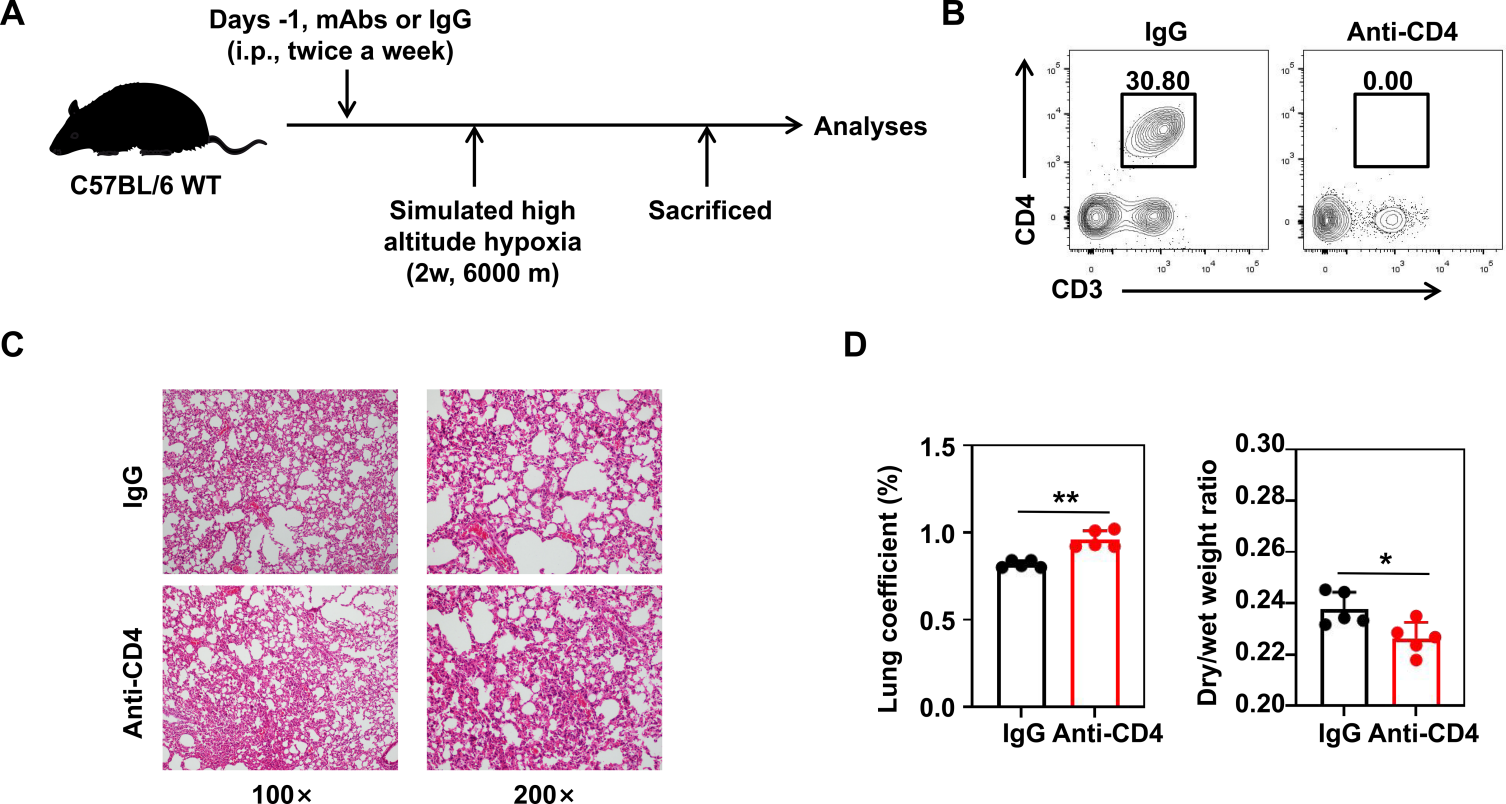
CD4 depletion promote the progression of high-altitude pulmonary edema. **(A)** Establishment of the CD4 depletion protocol in HH mice. **(B)** Verification of CD4 depletion efficiency. **(C)** HE staining of lung tissue sections in HH mice after 2 weeks of IgG and anti-CD4 treatment. **(D)** Lung coefficient and dry/wet weight ratio of HH mice after 2 weeks of IgG and anti-CD4 treatment. All data are presented as mean ± SD. *P < 0.05, **P < 0.01, n.s., *P* > 0.05.

Although we have confirmed that DCs in HH can induce CD4^+^ T cell immunosuppression, it remains unclear whether CD4^+^ T cells can alleviate the progression of HAPE. Therefore, we established a HAPE mouse model and depleted CD4^+^ T cells in the mice according to the protocol (**Figures 7A, B**). Pathological staining revealed that CD4 depletion exacerbated the progression of HAPE (**Figure 7C**). Moreover, the lung coefficient of HH mice with CD4 depletion significantly increased, and the dry/wet weight ratio of the lungs significantly decreased (**Figure 7D**).

## Discussion

The occurrence of HAPE involves multiple mechanisms. Blunted hypoxic ventilation response, pulmonary hypertension, and vasodilator (nitric oxide)/vasoconstrictor muscle imbalance is considered to be among the factors contributing to the formation of HAPE (34). However, more and more evidence indicates that the immune system plays a crucial role in the progression of HAPE. Studies have found that the absolute number of neutrophils and alveolar macrophages in bronchoalveolar lavage fluid (BALF) of patients with HAPE increases, and the levels of IL-1β, IL-6, IL-8 and TNF-α are significantly elevated (35–38). These changes lead to an immune imbalance in the lungs, a shift towards an inflammatory state, and cause high permeability of the alveolar capillary membrane (2, 33, 34, 39). Therefore, suppressing inflammation may be a potential approach for the treatment of HAPE.

Dendritic cells (DCs) originate from CD34^+^ bone marrow resident hematopoietic stem cells and are strategically distributed throughout the body, serving as a critical link between the innate and adaptive immune systems (40–42). In the microenvironment, cell interactions, cytokines, nutrients, metabolites, pH and oxygen (O_2_) levels jointly affect cellular behavior and function (43–45). O_2_ plays a pivotal role in shaping the metabolic and functional states of DCs, and a reduction in its concentration enables DCs to be programmed toward a tolerogenic phenotype (46). Moreover, inflammation can create a hypoxic environment in tissues, and in turn, hypoxia may also trigger inflammation (47, 48). This mechanism further aggravates hypoxia in the inflamed tissues. Therefore, the lung DCs of HAPE patients are more likely to perform immunosuppressive functions, thereby maintaining the immune homeostasis in the lungs as much as possible. This study shows that the proportion and absolute number of lung DCs in HH rats are significantly increased. Further analysis reveals that DCs in hypobaric hypoxic lungs tend to be in an immunosuppressive state, and its metabolic level changes significantly. Furthermore, our research found that the expression of HIF-1α in hypoxic DCs was upregulated, and its conditional deletion enhanced the pro-inflammatory activity of dendritic cells in the bacterial infection model (49). These results emphasize that hypoxic DCs can alleviate the progression of HAPE to a certain extent.

Kohler et al. demonstrated that hypoxia differentially regulates chemokine expression in bone marrow-derived dendritic cells via a HIF-1-dependent mechanism, increasing CCR7 expression and promoting the production of CCL17 and CCL22 (50). Moreover, when immature dendritic cells derived from human monocytes were incubated with IL-4 and granulocyte-macrophage colony-stimulating factor, and then exposed to an environment with 1% O_2_ for 4 days, there was minimal effect on the dendritic cell differentiation (51, 52). This exposure instead induces more migratory phenotypes, characterized by increased expression of the chemokine receptor genes CCR3, CCR2, and CX3CR1 (51). Here, our research results support the aforementioned conclusion that exposure to hypobaric hypoxia has a relatively minor effect on the regulation of Th2-type immune responses by dendritic cells, while the expression of genes related to maturation (CCL17, CCL22, Tnfrsf4, Stat6, Bcl2l1) and migration (CCR7, Myo1g, Fscn1) is altered. Furthermore, we observed that the gene expression profiles of different subgroups of dendritic cells varied under hypobaric hypoxic conditions, suggesting that they might perform distinct functions under such circumstances. In fact, a study has shown that cDC2 can be further divided into two groups: CD103^+^ and CD103^-^ (53). Among them, CD103^-^cDC2 has enhanced inflammatory potential (53). This indicates that in the lung of rats under hypobaric hypoxic conditions, there are still both pro-inflammatory and anti-inflammatory DCs, which may play different roles in the progression of HAPE.

Interestingly, a study found that hypoxia can promote the accumulation of CD4^+^ T cells in the lungs, which is consistent with our research results (54). Moreover, we also observed that lung T cells (especially CD4^+^ T cells) in HH rats produced more IL-10 and less TNF-α, suggesting that CD4^+^ T cells may contribute to the inhibition of HAPE progression. A number of studies have demonstrated that hypoxia can reduce the immune response of T cells by activating HIF-1α and restricting the stimulation of T cells by dendritic cells (49, 55, 56). In addition, hypoxia decrease T cell proliferation, promotes glycolysis, and leads to the emergence of IL-10^+^ Th17 and PD-1 and IL-10-secreting T cells population (57, 58). Here, we believe that the immunosuppression of T cells in the lungs of HH rats is mediated by DCs. Subsequent experiments confirmed our conjecture. Furthermore, hypoxic can also inhibit the maturation of DCs by inducing the expression of vascular endothelial growth factor (VEGF) (59, 60). VEGF in turn enhances the expression of programmed cell death ligand 1 (PD-L1) in dendritic cells and reduces T cell function (61, 62). Therefore, hypoxia can regulate DCs through different pathways and profoundly affect T cell function. This further suggests the possibility of using DC-T cell interaction to treat HAPE.

TNF-α is a pleipotent cytokine produced by activated macrophages, T cells and natural killer cells, and it is one of the most important regulatory factors of immune responses (63). Several studies have shown that TNF-α is elevated in pulmonary edema or lung injury induced by different factors (64–66). It can induce apoptosis of lung endothelial cells and damage the barrier function, thereby aggravating pulmonary edema (33, 67). When HAPE is relieved, the level of TNF-α also decreases, suggesting a correlation between TNF-α and the progression of HAPE (9, 64, 68). Our data further confirm that TNF-α can promote the progression of HAPE. Interestingly, our data show that when the total TNF-α level in rats increases, the number of DCs and CD4^+^ T cells decreases. A related study shows that the fewer these cell types there are, the more severe the acute mountain sickness will be (69). Strikingly, we also found that after TNF-α was injected into rats, the percentage, absolute number and proliferation ability of lung CD4^+^CD25^+^ T cells decreased. In addition, the level of TNF-α produced by CD4^+^ T cells was reduced. These data indicate that DCs and CD4^+^ T cells play a protective role in HAPE. They can prevent the further aggravation of HAPE. Furthermore, when TNF-α was deficient, the percentage and absolute number of DCs significantly increased, while the percentages of IL-6^+^ DC and IL-10^+^CD4^+^ T cells significantly decreased, indicating that TNF-α deficiency alleviated HAPE. Numerous studies have shown that TNF-α may affect the functions of DCs and T cells through multiple pathways (1): TNF-α directly binding to TNFR on DCs or T cells, thereby affecting their functions (70–72); (2) TNF-α indirectly affects the functions of DCs and T cells by influencing the differentiation of other cells (73, 74). Here, we and a previously study ruled out that CD4^+^ T cells and natural killer T (NKT) cells are not strong producers of TNF-α in HAPE. Therefore, hypoxia may induce macrophages to produce TNF-α, thereby promoting the progression of HAPE. Further analysis is expected to reveal the potential of regulating macrophage differentiation as a treatment for HAPE.

As pivotal regulators of adaptive immune responses, CD4^+^ T cells exhibit complex double-edged characteristics in acute lung injury (ALI) (75–77). In a mouse model of ALI, the blocking of Treg cells weakened the protective effect of berberine on lung injury, directly confirming the protective effect of Treg cells (75). In-depth research has revealed the functional heterogeneity of the CD4^+^ T cell population: Th1/Th17 cells destroy the integrity of alveolar structure by secreting inflammatory factors, while Treg cells alleviate lung injury by suppressing immune responses (77–79). This “pro-inflammatory *vs*. anti-inflammatory” bidirectional balance determines the progression of the disease. This study clarifies the crucial role of CD4^+^ T cells in the pulmonary microenvironment of HH rats, providing novel perspective for overcoming current therapeutic challenges of HAPE. Here, we found that CD4^+^ T cells produced less TNF-α and more IL-10 in the HH rat lungs, and further confirmed that the depletion of CD4^+^ T cells would lead to an accelerated progression of HAPE. Therefore, we believe that in the absence of intervention from other factors, CD4^+^ T cells in HAPE patients mainly play a protective role.

In conclusion, our research indicates that lung DCs induce CD4^+^ T cell immunosuppression in HH rats, thereby preventing excessive progression of HAPE. The dynamic changes in DCs and T cell subsets revealed that HAPE was alleviated after TNF-α deficiency. Moreover, the depletion of CD4^+^ T cells further confirmed its protective effect in HAPE. This study adds a new concept-immunotherapy-to the therapeutic paradigm of HAPE from “drug and physical therapy”, and provides key theoretical support for it. However, this study was conducted exclusively in animal models and did not include experiments with human specimens. Consequently, these findings may not fully reflect the data from human diseases.

## Data Availability

The data presented in the study are deposited in the NCBI repository, accession number PRJNA1422438.
