# Supplementary material for: Hypobaric hypoxia can lead to an increase in lung dendritic cells and promote T-cell immunosuppression, thereby preventing the excessive progression of high-altitude pulmonary edema
Source: Front Immunol. 2026 Mar 24;17:1752864. doi: 10.3389/fimmu.2026.1752864 (PMC13053275; doi:10.3389/fimmu.2026.1752864)
Supplement: Supplementary file 1 [file DataSheet1.doc]

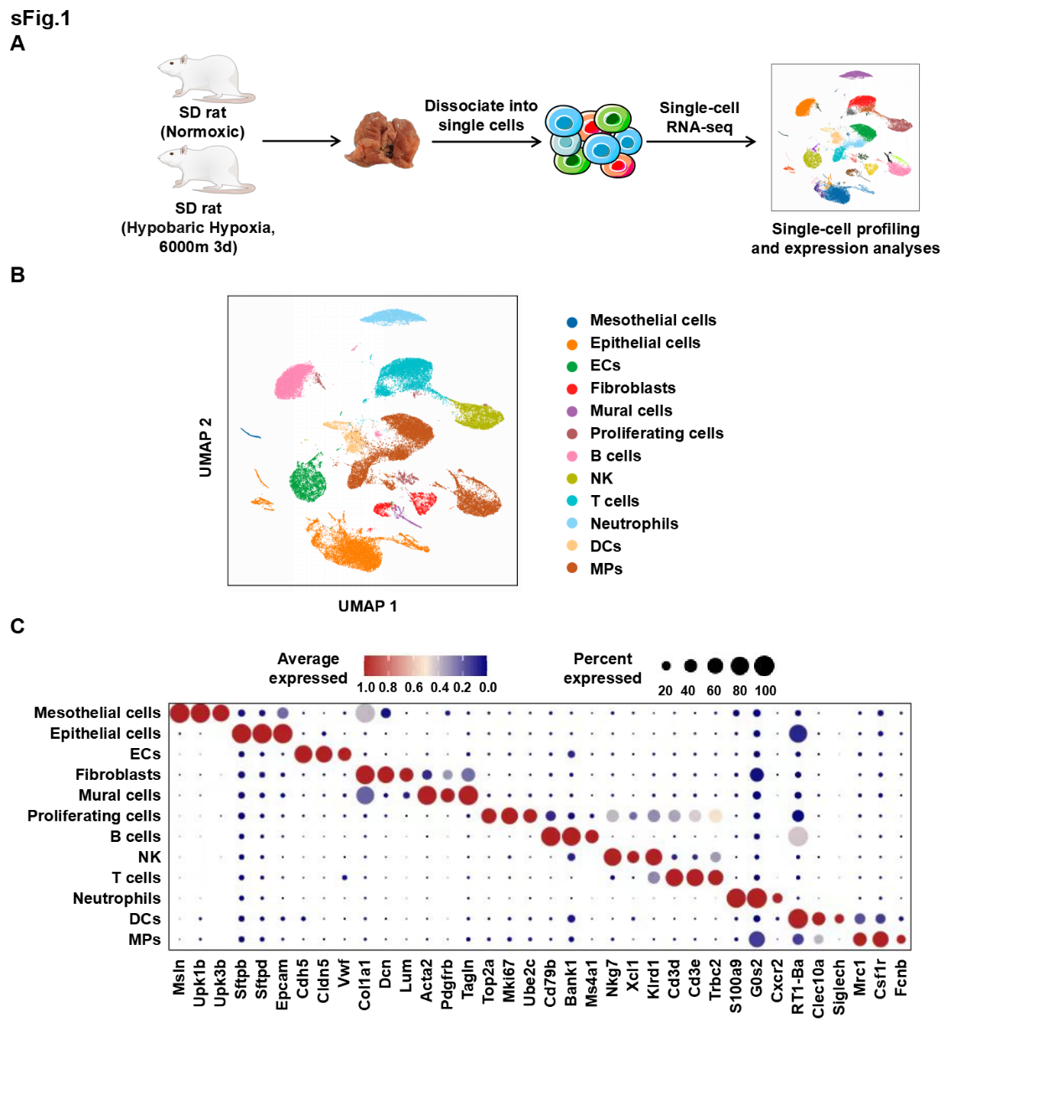


**Supplementary Figure 1. Single-cell sequencing of rat lungs and clustering analysis of different cell subpopulations**

**(A)** Schematic representation of single-cell sequencing experimental strategy. **(B)** UMAP plot depicts distribution of color-coded different cell populations as indicated. **(C)** The bubble chart shows the marker genes expressed by different cell populations in the rat lungs.


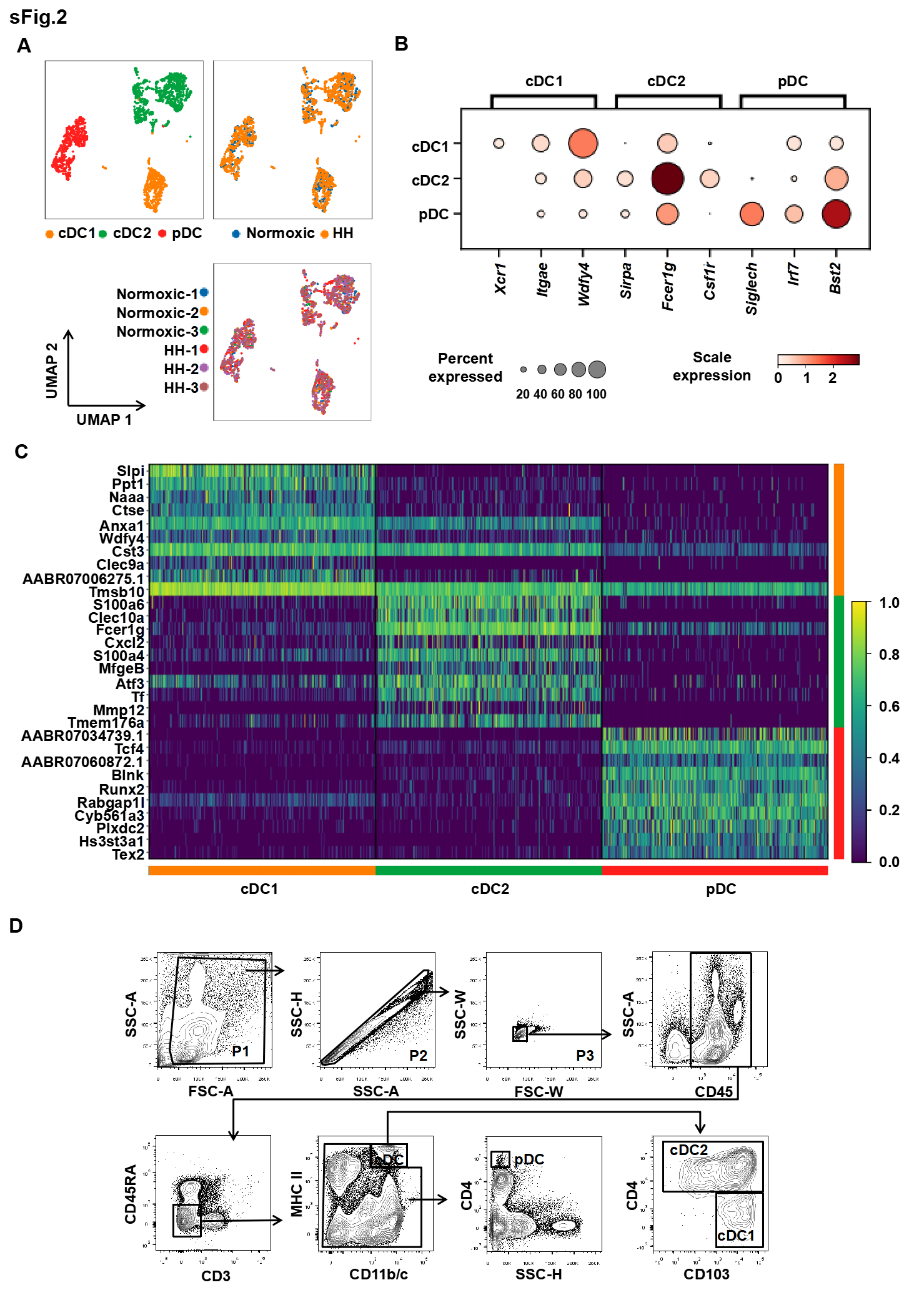


**Supplementary Figure 2. Cluster of rat lung DC subsets.**

**(A)** UMAP plot depicts distribution of color-coded DC clusters as indicated. **(B)** The bubble chart shows the marker genes expressed by cDC1, cDC2 and pDC in the rat lungs. **(C)** Heatmap shows the top 10 genes expressed in cDC1, cDC2 and pDC. **(D)** Gating strategy for flow cytometry analysis of rat lung DC subsets.


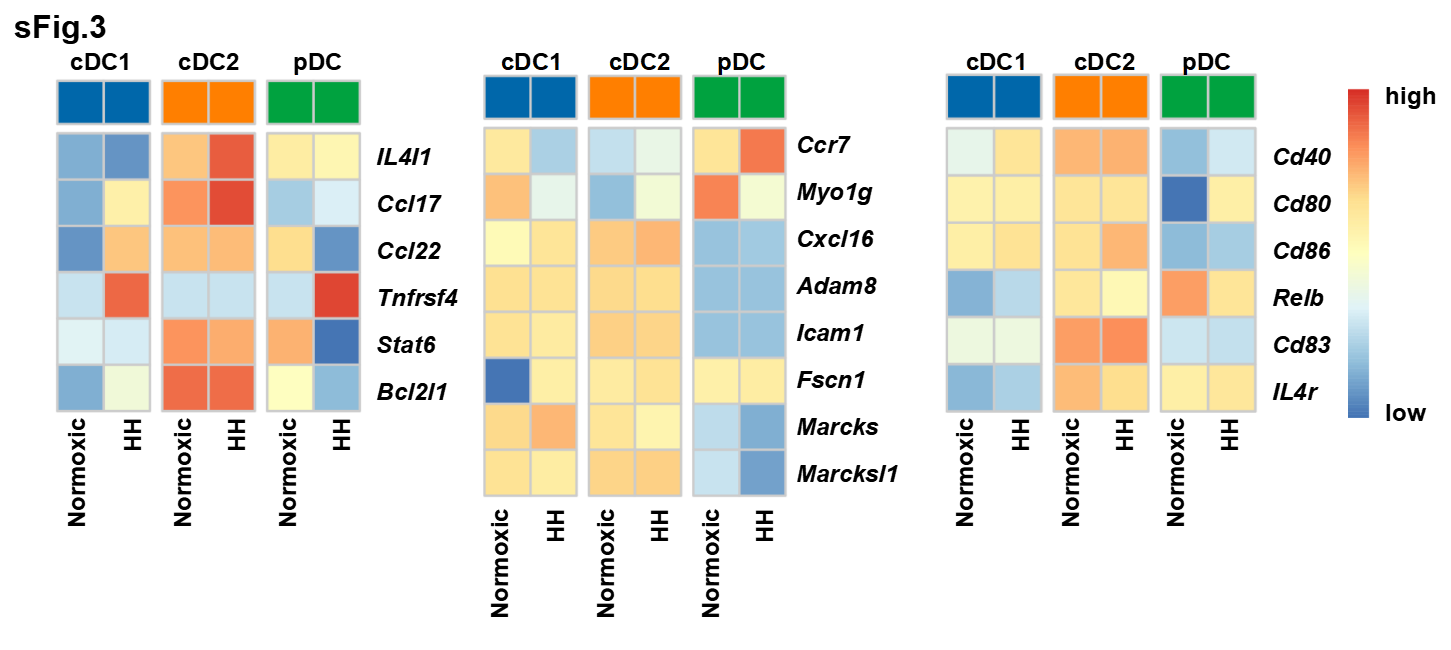


**Supplementary Figure 3. Heatmap displaying expression level of genes related to maturation, migration and Th2 response in the lung DC subsets of hypobaric hypoxia and normoxic rats.**


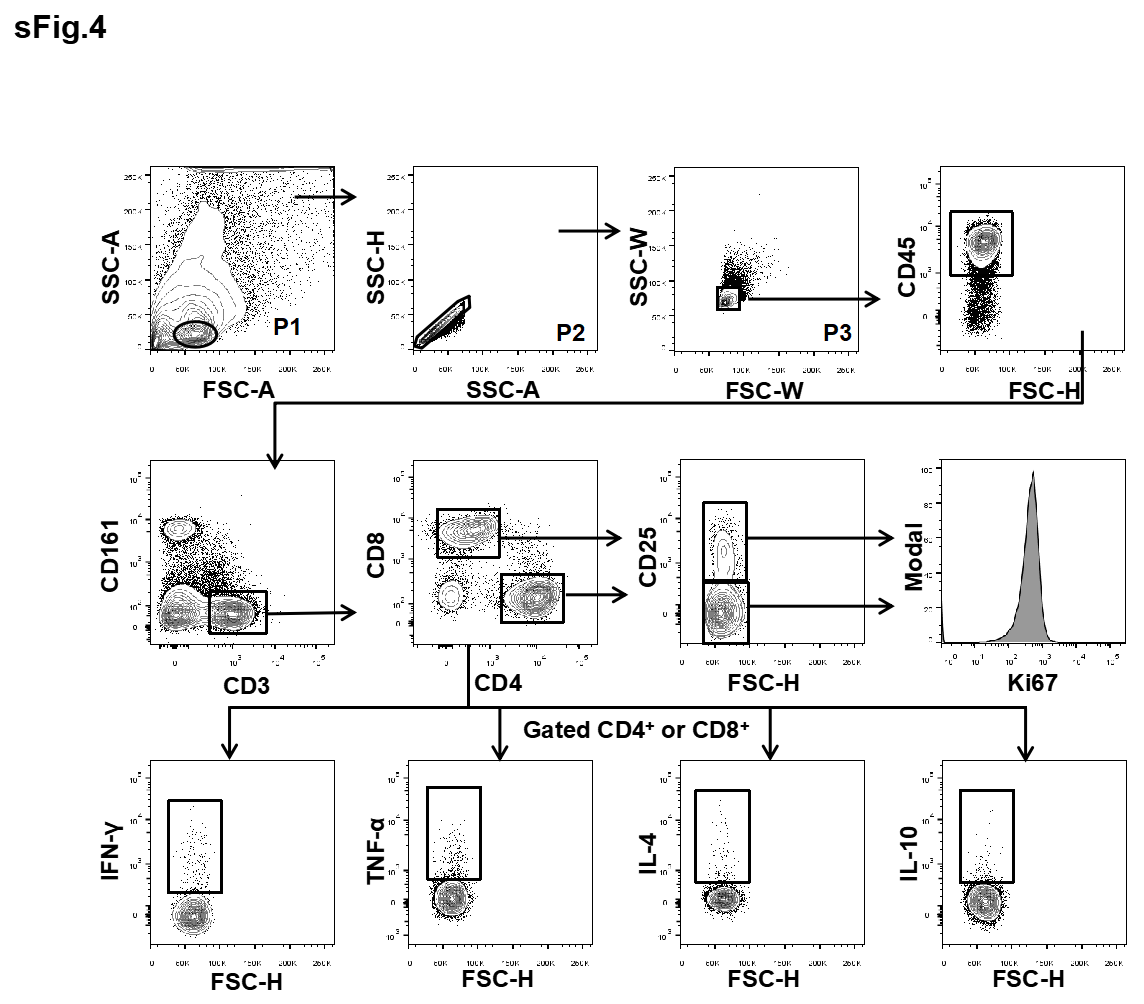


**Supplementary Figure 4.** **Gating strategy for flow cytometry analysis of rat lung T cells.**


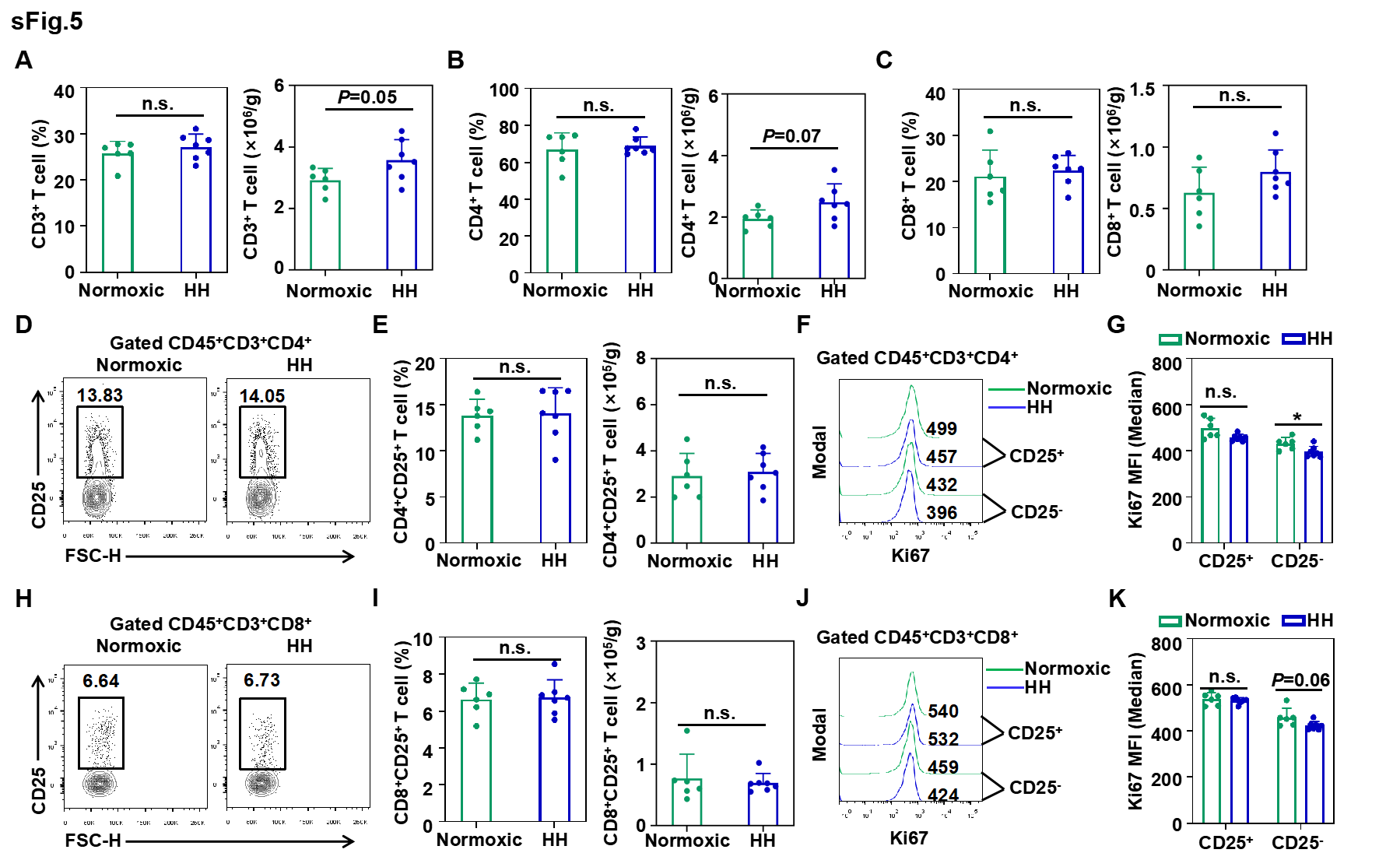


**Supplementary Figure 5. Hypobaric hypoxia did not change the phenotype of T cells in the rat lungs.**

**(A)** Percentage and absolute numbers of CD3^+^ T cells in the lung from normoxic and hypobaric hypoxia rats (6-7 rats per group). **(B)** Percentage and absolute numbers of CD4^+^ T cells in the lung from normoxic and hypobaric hypoxia rats (6-7 rats per group). **(C)** Percentage and absolute numbers of CD8^+^ T cells in the lung from normoxic and hypobaric hypoxia rats (6-7 rats per group). **(D)** Representative flow cytometry plot of CD4^+^CD25^+^ T cells in the lung from normoxic and hypobaric hypoxia rats. **(E)** Percentage and absolute numbers of CD4^+^CD25^+^ T cells in the lung from normoxic and hypobaric hypoxia rats (6-7 rats per group). **(F)** Representative flow cytometry plot of Ki67 expression in CD4^+^CD25^+^ and CD4^+^CD25^-^ T cells in the lung from normoxic and hypobaric hypoxic rats. **(G)** MFI of Ki67 expression in CD4^+^CD25^+^ and CD4^+^CD25^-^ T cells in the lung from normoxic and hypobaric hypoxic rats (6-7 rats per group). **(H)** Representative flow cytometry plot of CD8^+^CD25^+^ T cells in the lung from normoxic and hypobaric hypoxia rats. **(I)** Percentage and absolute numbers of CD8^+^CD25^+^ T cells in the lung from normoxic and hypobaric hypoxia rats (6-7 rats per group). **(J)** Representative flow cytometry plot of Ki67 expression in CD8^+^CD25^+^ and CD8^+^CD25^-^ T cells in the lung from normoxic and hypobaric hypoxic rats. **(K)** MFI of Ki67 expression in CD8^+^CD25^+^ and CD8^+^CD25^-^ T cells in the lung from normoxic and hypobaric hypoxic rats (6-7 rats per group). All data are presented as mean ± SD. **P* < 0.05, n.s., *P* > 0.05.


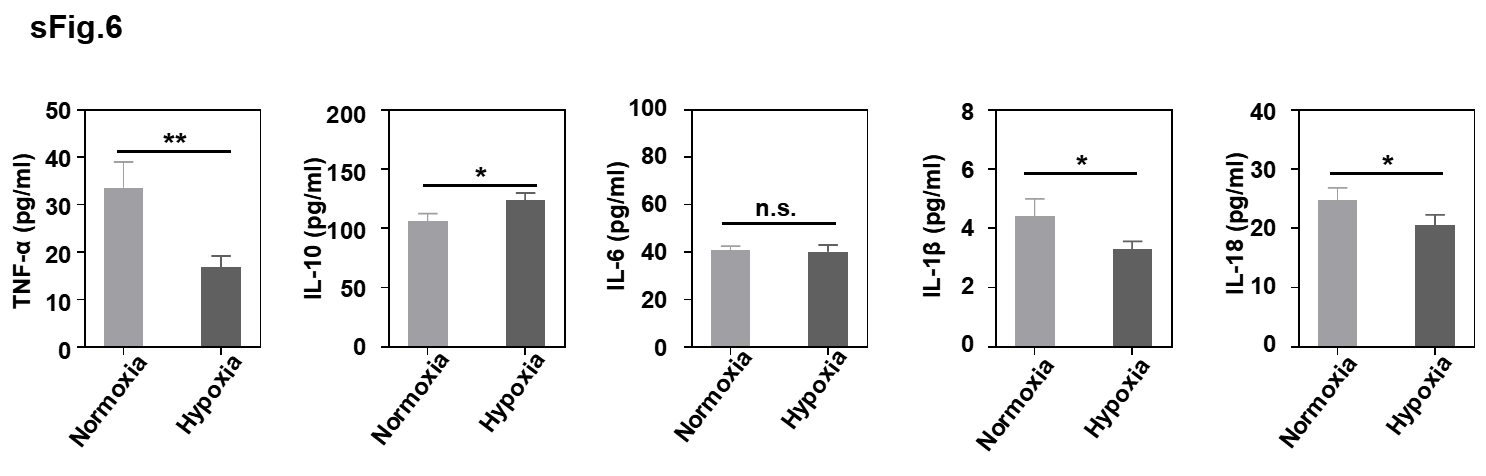
 **Supplementary Figure 6. TNF-α, IL-10, IL-6, IL-1β and IL-18 secretion in the supernatant of DC cocultured with T cells for 6 h in the presence of the normoxic or hypoxic was measured by ELISA.**


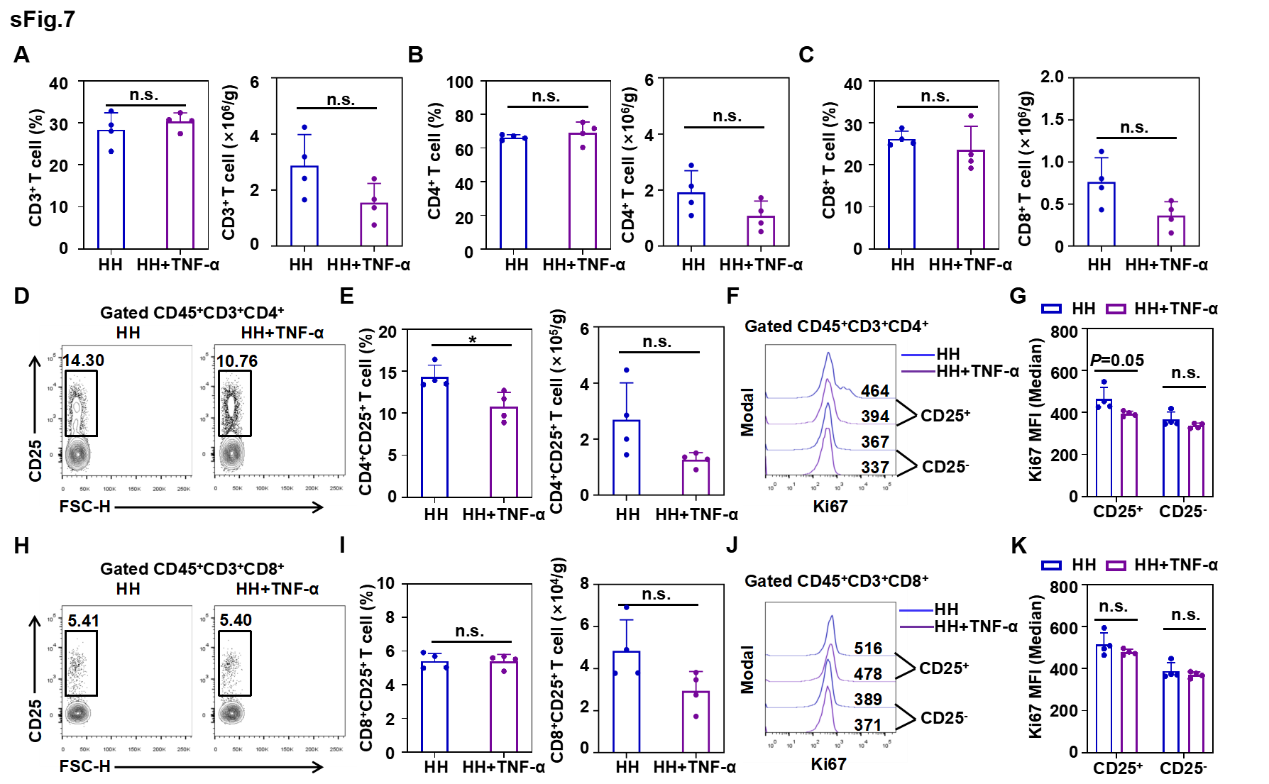


**Supplementary Figure 7. TNF-α inhibits the differentiation of CD4^+^CD25^+^ T cells in the lung of hypobaric hypoxic rats.**

**(A)** Percentage and absolute numbers of CD3^+^ T cells in the lung from hypobaric hypoxia and hypobaric hypoxia+TNF-α rats (4 rats per group). **(B)** Percentage and absolute numbers of CD4^+^ T cells in the lung from hypobaric hypoxia and hypobaric hypoxia+TNF-α rats (4 rats per group). **(C)** Percentage and absolute numbers of CD8^+^ T cells in the lung from hypobaric hypoxia and hypobaric hypoxia+TNF-α rats (4 rats per group). **(D)** Representative flow cytometry plot of CD4^+^CD25^+^ T cells in the lung from hypobaric hypoxia and hypobaric hypoxia+TNF-α rats. **(E)** Percentage and absolute numbers of CD4^+^CD25^+^ T cells in the lung from hypobaric hypoxia and hypobaric hypoxia+TNF-α rats (4 rats per group). **(F)** Representative flow cytometry plot of Ki67 expression in CD4^+^CD25^+^ and CD4^+^CD25^-^ T cells in the lung from hypobaric hypoxia and hypobaric hypoxia+TNF-α rats. **(G)** MFI of Ki67 expression in CD4^+^CD25^+^ and CD4^+^CD25^-^ T cells in the lung from hypobaric hypoxia and hypobaric hypoxia+TNF-α rats (4 rats per group). **(H)** Representative flow cytometry plot of CD8^+^CD25^+^ T cells in the lung from hypobaric hypoxia and hypobaric hypoxia+TNF-α rats. **(I)** Percentage and absolute numbers of CD8^+^CD25^+^ T cells in the lung from hypobaric hypoxia and hypobaric hypoxia+TNF-α rats (4 rats per group). **(J)** Representative flow cytometry plot of Ki67 expression in CD8^+^CD25^+^ and CD8^+^CD25^-^ T cells in the lung from hypobaric hypoxia and hypobaric hypoxia+TNF-α rats. **(K)** MFI of Ki67 expression in CD8^+^CD25^+^ and CD8^+^CD25^-^ T cells in the lung from hypobaric hypoxia and hypobaric hypoxia+TNF-α rats (4 rats per group). All data are presented as mean ± SD. **P* < 0.05, n.s., *P* > 0.05.


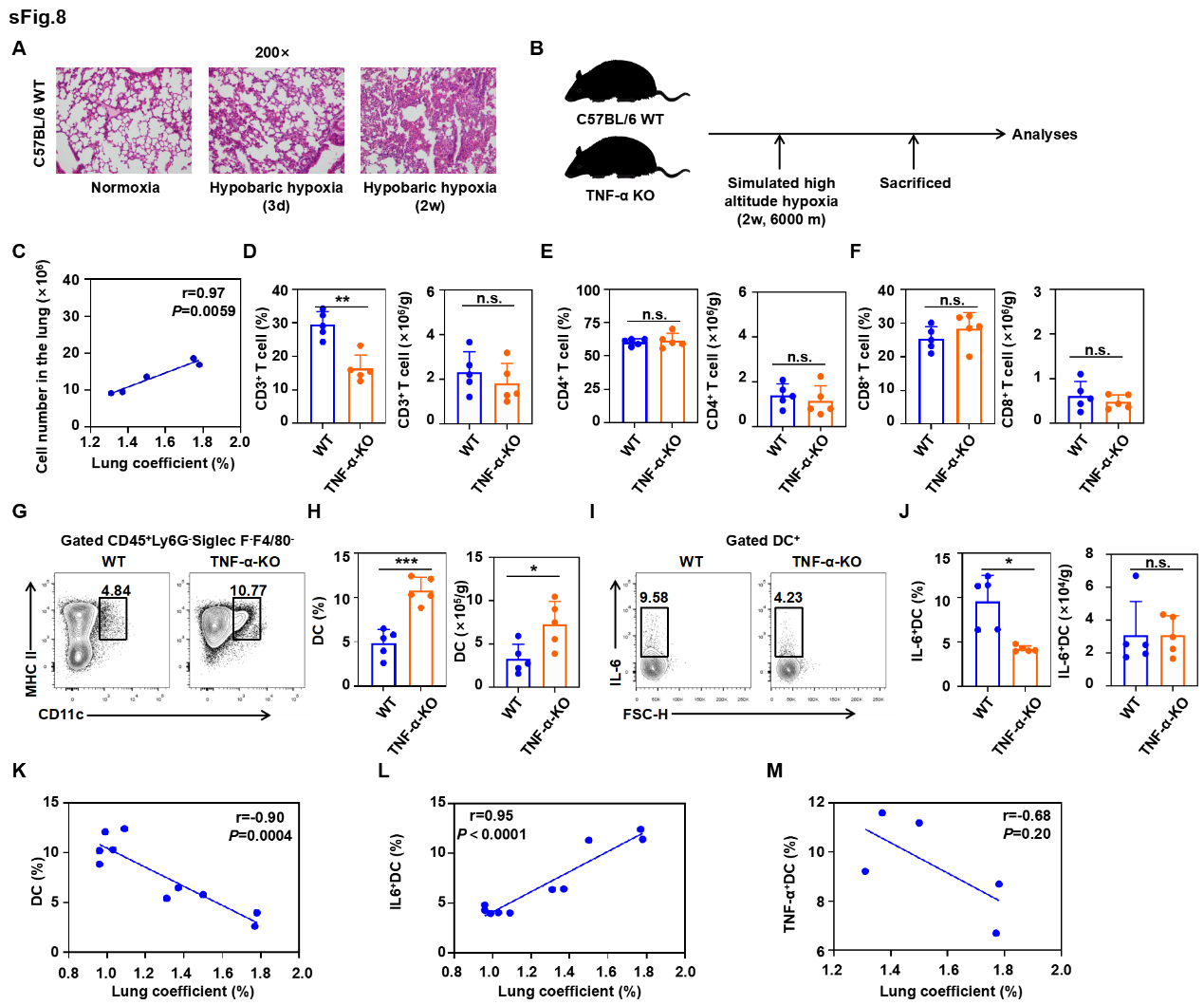


**Supplementary Figure 8.** **TNF-α deficiency can increase lung DCs in HH mice.**

**(A)** Representative HE staining images of lung sections in WT mice with normoxia or hypoxic hypoxia for 3 d and 2 weeks. **(B)** Establishment of a HAPE model in mice induced by hypoxic hypoxia. **(C)** Correlation between the lung coefficient and the cell number in the lung from WT mice after 2 weeks of hypobaric hypoxia. **(D)** Percentage and absolute numbers of CD3^+^ T cells in the lung from WT and TNF-α-KO mice after 2 weeks of hypobaric hypoxia (5 mice per group). **(E)** Percentage and absolute numbers of CD4^+^ T cells in the lung from WT and TNF-α-KO mice after 2 weeks of hypobaric hypoxia (5 mice per group). **(F)** Percentage and absolute numbers of CD8^+^ T cells in the lung from WT and TNF-α-KO mice after 2 weeks of hypobaric hypoxia (5 mice per group). **(G)** Representative flow cytometry plot of DC (CD45^+^Ly6G^-^Siglec F^-^F4/80^-^CD11c^+^MHC Ⅱ^+^) in the lung from WT and TNF-α-KO mice after 2 weeks of hypobaric hypoxia. **(H)** Percentage and absolute numbers of DC in the lung from WT and TNF-α-KO mice after 2 weeks of hypobaric hypoxia (5 mice per group). **(I)** Representative flow cytometry plot of IL-6 production by DC in the lung from WT and TNF-α-KO mice after 2 weeks of hypobaric hypoxia. **(J)** Percentage and absolute numbers of IL-6 production by DC in the lung from WT and TNF-α-KO mice after 2 weeks of hypobaric hypoxia (5 mice per group). **(K)** Correlation between the lung coefficient and the percentage of lung DC from WT and TNF-α-KO mice after 2 weeks of hypobaric hypoxia (5 mice per group). **(L)** Correlation between the lung coefficient and the percentage of IL-6 produced by lung DC from WT and TNF-α-KO mice after 2 weeks of hypobaric hypoxia (5 mice per group). **(M)** Correlation between the lung coefficient and the percentage of TNF-α produced by lung DC from WT mice after 2 weeks of hypobaric hypoxia. All data are presented as mean ± SD. **P* < 0.05, ***P* < 0.01, ****P* < 0.001, n.s., *P* > 0.05.
