# Supplementary material for: Hypobaric hypoxia can lead to an increase in lung dendritic cells and promote T-cell immunosuppression, thereby preventing the excessive progression of high-altitude pulmonary edema
Source: Front Immunol. 2026 Mar 24;17:1752864. doi: 10.3389/fimmu.2026.1752864 (PMC13053275; doi:10.3389/fimmu.2026.1752864)
Supplement: Supplementary file 2 [file Table1.doc]

**Supplemental Table 1. Antibodies for flow cytometry**

| **Reagent or Resource** | **Clone** | **Source** | **Catalog Number** |
| --- | --- | --- | --- |
| Purified anti-mouse CD16/32 |  | BioLegend | Cat# 101302 |
| Anti-rat CD3-PE-Cy7 | 1F4 | BioLegend | Cat# 201421 |
| Anti-rat CD161-FITC | 3.2.3 | BioLegend | Cat# 205608 |
| Anti-rat CD4-Brilliant Violet 605 | OX-38 | BD | Cat# 743090 |
| Anti-rat CD8-Brilliant Violet 510 | OX-8 | BD | Cat# 740139 |
| Anti-rat CD25-APC | OX-39 | BioLegend | Cat# 202114 |
| Anti-rat Ki67-Brilliant Violet 510 | B56 | BD | Cat# 563462 |
| Anti-rat IFN-γ-Alexa Fluor 647 | DB-1 | BioLegend | Cat# 507810 |
| Anti-rat IL-10-PE | A5-4 | BD | Cat# 555088 |
| Anti-mouse/rat TNF-α-APC | TN3-19.12 | BioLegend | Cat# 506108 |
| Anti-rat IL-4-PE | OX-81 | BioLegend | Cat# 511906 |
| Anti-rat CD11b/c-FITC | OX-42 | BioLegend | Cat# 201805 |
| Anti-rat RT1B-PE | OX-6 | BioLegend | Cat# 205308 |
| Anti-rat CD103-Alexa Fluor 647 | OX-62 | BioLegend | Cat# 205509 |
| Anti-rat CD45RA-PerCP/Cy5.5 | OX-33 | BioLegend | Cat# 202318 |
| Anti-rat CD45-Alexa Fluor 700 | OX-1 | BioLegend | Cat# 202218 |
| Anti-mouse CD45-PerCP/Cy5.5 | S18009F | BioLegend | Cat# 157208 |
| Anti-mouse CD11b-Brilliant Violet 605 | M1/70 | BioLegend | Cat# 101237 |
| Anti-mouse Ly6G-Alexa Fluor 700 | 1A8 | BioLegend | Cat# 127621 |
| Anti-mouse siglec-F-Brilliant Violet 421 | S17007L | BioLegend | Cat# 155532 |
| Anti-mouse Ly6C-Pacific Blue | HK1.4 | BioLegend | Cat# 128013 |
| Anti-mouse F4/80-FITC | BM8 | BioLegend | Cat# 123107 |
| Anti-mouse MHC Ⅱ-APC | 34-1-2S | BioLegend | Cat# 114713 |
| Anti-mouse CD11c-Brilliant Violet 510 | N418 | BioLegend | Cat# 117337 |
| Anti-mouse IL-6-PE | MP5-20F3 | BioLegend | Cat# 504504 |
| Anti-mouse TNF-α-PE/Dazzle 594 | MP6-XT22 | BioLegend | Cat# 506346 |
| Anti-mouse IL-10-PE/Dazzle 594 | JES5-16E3 | BioLegend | Cat# 505034 |
| Anti-mouse CD3-FITC | 17A2 | BioLegend | Cat# 100204 |
| Anti-mouse NK1.1-PE-Cy7 | S17016D | BioLegend | Cat# 156514 |
| Anti-mouse CD4-Brilliant Violet 605 | GK1.5 | BioLegend | Cat# 100451 |
| Anti-mouse CD8-APC/Cy7 | YTS156.7.7 | BioLegend | Cat# 126619 |
| Anti-mouse CD25-Pacific Blue | PC61 | BioLegend | Cat# 102022 |
